# BaDuShengJi hydrogel accelerates diabetic wounds healing and regeneration of deep burn injury by anti-bacteria, anti-inflammation and promoting epithelialization

**DOI:** 10.3389/fphar.2025.1580994

**Published:** 2025-06-04

**Authors:** Yuxiang Zhang, Yuanyuan Yu, Lien Yu, Fang Wang, Fuwei Yang, Shiyu Liang, Wen Xu, Mengying Ji, Yinuo Geng, Jingwei Xue, Chunmao Han, Zhongtao Zhang, Yilin Zhang

**Affiliations:** ^1^ Department of Burns and Wound Care Center, Second Affiliated Hospital, College of Medicine, Zhejiang University, Hangzhou, China; ^2^ Tumor Precise Intervention and Translational Medicine Laboratory, The Affiliated Taian City Central Hospital of Qingdao University, Taian, China; ^3^ Shandong Provincial Key Medical and Health Laboratory of Microenvironment-Responsive Biomedical Materials, Taian City Central Hospital, Taian, China; ^4^ Department of Nursing Division, First Affiliated Hospital, College of Medicine, Zhejiang University, Hangzhou, China; ^5^ Department of Nursing Division, Second Affiliated Hospital, College of Medicine, Zhejiang University, Hangzhou, China; ^6^ Department of Nursing, Taishan Vocational College of Nursing, Taian, China; ^7^ Taishan Academy of Medical Sciences, The Affiliated Taian City Central Hospital of Qingdao University, Taian, China; ^8^ Postdoctoral Mobile Station, Shandong University of Traditional Chinese Medicine, Jinan, China; ^9^ Affiliated Hospital of Shandong Institute of Traditional Chinese Medicine, Jinan, China

**Keywords:** BaDuShengJi San, wound healing, diabetic wound, deep burn injury, anti-bacteria, anti‐inflammation, promote re-epithelialization

## Abstract

Bacterial infection, excessive inflammation, and delayed re-epithelialization are the biggest obstacles to diabetic wound healing and burn injuries. As a classic prescription, BaDuShengJi San (BDS) has been proven to accelerate chronic wound healing through anti-bacterial and anti-inflammatory mechanisms. However, there are few reports on its role in preventing the malignant progression of deep burns or accelerating deep burn wound healing. Furthermore, the powder formulation of BDS has limited application scenarios, difficult dosage control, potential environmental pollution, and contradiction with the theory of moist wound healing, which has constrained its scope of application and efficacy to some extent. To address these issues, this study developed a novel hydrogel formulation incorporating BDS using carbomer 940 as the gel matrix, glycerol as a humectant, and triethanolamine as a pH adjuster, through orthogonal design. The results demonstrated that the prepared BDS-loaded gel (BDN) exhibited excellent stability and good biocompatibility, preserving the antibacterial and angiogenesis-promoting properties of BDS while enhancing its anti-inflammatory capabilities. Additionally, BDN significantly accelerated re-epithelialization in diabetic chronic wounds and burn wounds compared to BDS, achieving superior healing quality and mitigating renal damage associated with long-term BDS application. This study provides a comprehensive strategy for accelerating wound healing in diabetic chronic wounds and deep burn injuries and offers insights into expanding the clinical application of BDS and enhancing its therapeutic effects.

## 1 Introduction

The prevalence of wounds poses a significant threat to human health, resulting in considerable economic burdens (Freedman et al., 2023). As the population ages and obesity rates rise, the need for effective wound management strategies has become increasingly urgent, particularly for diabetic chronic wounds. Studies have shown that 25% of diabetic patients develop chronic wounds, which are associated with higher rates of amputation and mortality (Sun et al., 2024; Yang et al., 2023; Theodore Hart et al., 2017; Armstrong et al., 2023). This underscores the importance of developing innovative and efficient wound healing treatments.

The standard clinical approach to wound treatment involves surgical debridement followed by hemostasis. The healing process is complex, encompassing inflammation, proliferation, and remodeling phases (Tang et al., 2023). However, delayed re-epithelization, excessive inflammatory reaction, and bacterial infection are the primary factors that hinder wound closure in deep burn injury and diabetic chronic wounds (Zhu S. et al., 2023; Xu et al., 2021; Zhong et al., 2022).

Re-epithelization is crucial for reconstructing the epidermal layer, involving the separation, proliferation, migration, and differentiation of epidermal keratinocytes (Martin and Nunan, 2015; Singer and Clark, 1999). This process is vital for restoring the skin’s barrier function, which is essential for maintaining internal stability and defending against external threats. In diabetic patients, hyperglycemia disrupts this process, leading to impaired angiogenesis, re-epithelialization, and collagen deposition (Armstrong et al., 2017). Due to the hyperglycemic effects associated with diabetes, wound healing in diabetic patients is characterized by lack of coordination and spatial disorganization compared to normal healing (Shao et al., 2023). Manifestations include impaired angiogenesis and re-epithelialization, degraded collagen deposition, and epithelial structural disorders (Armstrong et al., 2017). Deep burn wounds, often with extensive skin loss, face difficulties in achieving complete re-epithelization shortly and thus frequently require autologous/allogeneic skin grafting for wound coverage. However, limited skin donor sites and graft availability pose significant challenges (Wang et al., 2018; Jeschke et al., 2020; Derkenne et al., 2019).

The inflammatory phase is indispensable in wound repair, as it clears harmful substances, initiates immune responses, and provides a clean environment for tissue regeneration and wound healing (Singer and Clark, 1999; Pena and Martin, 2024). It also promotes the proliferation and differentiation of wound healing-related cells, such as fibroblasts, by releasing growth factors, creating conditions for subsequent granulation tissue formation and re-epithelization (Singer and Clark, 1999). However, persistent inflammation hinders the transition to the proliferative and tissue remodeling stages, leading to delayed wound healing and, even after healing, uneven wound surfaces and scarring (Martin and Nunan, 2015). Diabetic wounds experience chronic inflammation due to macrophage polarization dysregulation, imbalanced pro-inflammatory/anti-inflammatory cytokine expression, and increased oxidative stress, which exacerbates ischemia and hypoxia in the wound and leads to necrosis (Martin and Nunan, 2015; Chang and Nguyen, 2021; Sharifiaghdam et al., 2022; Huang et al., 2022). Deep second-degree burns, with damage to the deep dermis, often exhibit progressive worsening and require prompt surgical or medical intervention for healing. Studies have found that the post-burn inflammatory cascade leads to excessive release of inflammatory factors, causing tissue edema, ischemia, and progressive wound deepening or even necrosis (Fang et al., 2017). Therefore, controlling excessive inflammation is essential to prevent wound deterioration and accelerate healing.

Due to the loss of skin’s protective barrier, blood, exudate, and damaged tissues provide a nutrient basis for rapid bacterial growth. Bacterial infection not only enhances local inflammatory response, disrupts normal wound healing processes, but can also severely induce sepsis, threatening patients' lives (Uberoi et al., 2024). While antibiotics such as tetracycline, neomycin, and quinolones are currently used for wound healing, non-antibiotic antibacterial strategies are gaining attention due to antibiotic restrictions (Sun et al., 2024).

In summary, bacterial infection, wound inflammation, and re-epithelization are interconnected and mutually influential. Preventing wound infection, controlling excessive inflammation, and promoting re-epithelization through appropriate means can accelerate wound healing and restore skin function. Existing treatment strategies, including antibiotics (Chang and Nguyen, 2021; Liu et al., 2024), surgical debridement (Wang et al., 2018), skin grafting (Sun et al., 2014), as well as hyperbaric oxygen (Wunderlich et al., 2000), stem cells (Yu et al., 2023), growth factors (Desmet et al., 2018), have shown limited efficacy and are often costly. Therefore, there is an urgent need to develop a novel strategy for comprehensive wound treatment to accelerate the healing of chronic and burn wounds.

“BaDuShengJi San (BDS),” a traditional Chinese medicine preparation which consists of Red Ochre, Huangdan, Calomel, Calcined Calamine, Calcined Os Draconis, Insect-white Wax, Calcined Plaster, Borneol., has been documented for its detoxifying properties and promotion of tissue regeneration and wound healing in the Qing Dynasty’s “Secrets of Injury Treatment” (Cheng et al., 2023). Red Ochre and Huangdan are the monarch drugs, providing both cold and warm properties to aid in toxin expulsion and healing. Calomel acts as minister drug by promoting ulcer healing and removing necrotic tissue. Calcined Calamine absorbs dampness and prevents putrefaction, while Calcined Os Draconis stops bleeding and aids tissue regeneration. Calcined Plaster clears heat and promotes healing. Together, these ingredients act as guide drugs to reduce toxicity, and support the monarch drugs. Borneol has a pungent, bitter, cool, and clear nature. When used externally, it clears heat, alleviates pain, reduces swelling, and promotes tissue regeneration. Ceresin wax has a sweet, warm, and non-toxic nature, and excels at stopping bleeding and promoting tissue regeneration. When these two assistant drugs are combined, they assist the monarch and minister drugs in astringing wounds and promoting tissue regeneration.

BDS has demonstrated clear efficacy in expediting the healing process of chronic wounds, with numerous studies highlighting its application in the treatment of diabetic foot ulcers (Cui and Hu, 2024; Guo et al., 2018), non-lactational mastitis (Zhang et al., 2025; Xie and Dai, 2021; Cao et al., 2018), postoperative wounds associated with anorectal diseases (Wang, 2024; Xiang, 2021), refractory wounds following calcaneal fracture surgery (Yin, 2019). For example, the use of BDS, either alone or in conjunction with debridement techniques such as gradual debridement, can substantially reduce the treatment duration for diabetic foot ulcers (Cui and Hu, 2024; Guo et al., 2018). The Wnt/β-catenin and Keap1/Nrf2 pathways have been identified as being closely linked to its capacity to enhance the healing of diabetic ulcers (Wang and Li, 2023a; Wang and Li, 2023b). In cases with a high risk of postoperative wound infection and delayed wound healing, such as low perianal abscesses and simple anal fistulas, BDS can significantly decrease the length of hospital stay, mitigate postoperative pain, and markedly enhance patient satisfaction (Xiang, 2021; Chen and Zeng, 2024). Recent research has identified that the application of BDS-impregnated thread for ligation in the treatment of fistulas located in the areola region, associated with non-lactational mastitis, can significantly reduce the time required for fistula opening, expedite the healing process, and decrease both infection and recurrence rates (Zhang et al., 2025). In light of the notable efficacy of BDS in managing chronic wounds, an expert consensus on its clinical application was established in 2023, which aims to standardize the rational use of BDS in clinical practice and to provide guidance for the management of wound-related diseases utilizing BDS (Cheng et al., 2023).

The skin’s compromised barrier at the site of a burn or scald significantly elevates the risk of infectious complications. Moreover, the inflammatory cascade activated by such thermal trauma is a principal etiology in the progression of wounds, often culminating in deeper tissue injury and necrosis. Although BDS is currently utilized for various types of chronic refractory wounds, but its applications in preventing and treating acute wounds like deep burns has not been extensively reported. Modern research suggests that BDS can kill bacteria, promote granulation tissue growth, provide a favorable microenvironment for wound healing and epithelial repair, improve local tissue blood circulation, and exhibit anti-inflammatory effects (Cheng et al., 2023). These properties of BDS align with current strategies for preventing early deepening of burn wounds and accelerating their healing (Singh et al., 2007). Therefore, it is speculated that this formulation can alleviate the burn wound progressive deepening and promote the repair of deep second-degree burn wounds.

Despite the evident therapeutic effect of BDS on wounds, its clinical use as a powder formulation encounters several challenges. These include difficulty in reaching deep-seated ulcers, inconvenience in administration, large single-use dosages, the potential for heavy metal poisoning, and environmental pollution due to powder scattering (Liu et al., 2020). Furthermore, the powder formulation does not maintain a moist environment on the wound surface for an extended period, which is essential for wound healing (WINTER, 1962).

**SCHEME 1:**
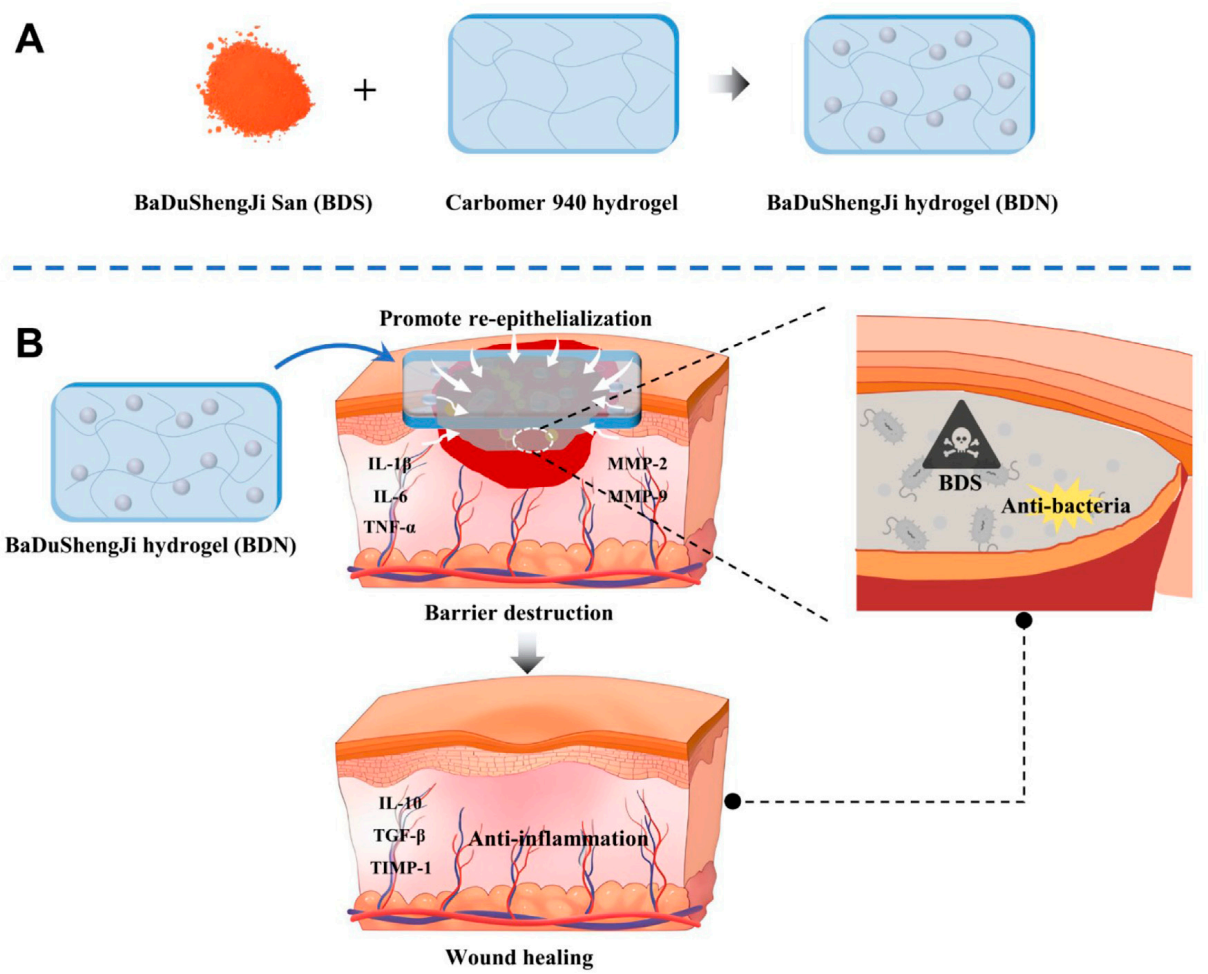
Schematic illustration for **(A)** the preparation of BaDuShengJi hydrogel and **(B)** the barrier destruction and promoting wound healing mechanisms of BDN. The picture was created by PowerPoint 2019 and Adobe illustrator 2024.

In this study, we have successfully reformulated BDS into a hydrogel, denoted as BaiDuShengJi gel (BDN), which introduces an array of advantageous attributes beyond those inherent to BDS. These improvements encompass enhanced mechanical plasticity, heightened adhesiveness, improved moisture retention capabilities, a potent drug depot function, and superior barrier properties. Furthermore, the meticulous preparation of BDN is anticipated to elevate both the safety profile and therapeutic efficacy of BDS in wound healing applications, thereby rendering it an even more viable candidate for clinical adoption (Scheme 1). The research not only broadens the clinical applicability of BDS in managing acute burn wounds and chronic diabetic wounds but also establishes a versatile framework for the modification of powder dosage forms. This approach offers a novel and promising strategy for optimizing therapeutic interventions in the realm of wound healing.

## 2 Materials and methods

### 2.1 Preparation and properties of hydrogels

#### 2.1.1 The screening of matrix for preparation of BDN

Various concentrations of carbomer 940, sodium alginate and CMC-Na were prepared using ultrapure water. Centrifugal stability, homogeneity, and fluidity were used to screen the gel matrices. In addition, experiments on the mixing and formation of BDN were conducted in this section. BDS suspensions were prepared using ultrapure water, followed by the preparation of 1% carbomer 940 gel, 10% sodium alginate gel, and 5% CMC-Na gel, each containing 1 mg/mL of the drug. The changes in gel state and the dispersion of the drug within the gel were recorded.

#### 2.1.2 The screening of humectant for preparation of BDN

Moisture retention was used to screen the optimal humectant. Two portions of the preferred BDN were prepared, and the same mass of 5% glycerol and 1,2-propanediol were added to each, respectively. The mixtures were thoroughly stirred and then transferred into Petri dishes with constant weight.

The moisture-retention Rate (%) was calculated as follows:

$$S_{i}=\left(M_{0}-M_{i}\right)\;/\;M_{s}\times 100\%;$$

$$\text{Moisture}-\text{retention Rate }\left(\%\right)=\left(1-S_{i}\right)\times 100\%$$

S_i_: Water loss rate at the time I, M_0_: Total weight before drying, M_i_: Total weight at the time i, M_s_: Weight of moisturizing agent and water.

#### 2.1.3 The orthogonal experimental design for the preparation of BDN

The content of carbomer 940, the dosage of BDS, pH value, and propylene glycol content in the gel were investigated, and the prescription of the gel was optimized by orthogonal design with the comprehensive sensory score as the evaluation criteria.

#### 2.1.4 Characterization of BDN

Use a Scanning Electron Microscope (SEM) (ZEISS ULTRATM 55) to observe the internal morphology and element mapping of various hydrogel samples. An advanced rotational rheometer (ARES G2, TA Instruments, USA) was used to perform oscillatory frequency scans of hydrogel samples at 24°C to analyze the gelation process.

Swelling ratio (%): Immerse lyophilized hydrogel sample (W_0_) in 5 mL PBS solution (pH = 7.4). At interval time point, blot excess surface water and weigh the swollen hydrogel (W_t_). Three parallel samples are used for each group. The swelling ratio (SR) is calculated by the following formula:

$$\text{SR }\left(\%\right)=\left(W_{t}-W_{0}\right)/W_{0}\times 100\%$$

### 2.2 *In vitro* antibacterial test

#### 2.2.1 Turbidity and plate count method

The Gram-positive bacteria *Staphylococcus aureus* (*S. aureus,* ATCC 8099) and the Gram-negative bacteria *Escherichia coli* (*E. coli,* ATCC 6538) were used. A total of 1 mL of bacteria (10^6^ CFU/mL for *S. aureus* and *E. coli*) were treated with different concentrations of BDN. The *in vitro* antimicrobial activity of BDN against *S. aureus* and *E. coli* was assessed using the turbidity and plate count method, live/dead staining for bacteria.

#### 2.2.2 Anti-biofilms effect of BDN

The anti-biofilm effect of BDN was studied using SEM and crystal violet staining. For biofilm culturing, an *S. aureus* suspension and Mueller-Hinton broth medium were placed in 96-well plates and cultured at 37°C for 24 h. The anti-biofilm effect of different concentrations of BDS and BDN extracts was incubated for 24 h. After treatment, 0.5% crystal violet solution was added to the biofilms, stained for 30 min, and washed 6 times with PBS. Lastly, the crystal violet was dissolved in methanol, and the absorbance at 595 nm was measured using a microplate reader. For SEM observation, glutaraldehyde and osmic acid solutions were used to fix the samples. Dehydrate the samples using an alcohol gradient (30%, 50%, 70%, 90%, 100%) and spray the surface with gold. Examine the anti-biofilm effect using an emission scanning electron microscope (Hitachi SU-8010).

### 2.3 Evaluation of anti-inflammatory performance of BDN on raw 264.7

RAW 264.7 was incubated in a 6-well plate for 24 h, followed by co-incubation with LPS and various samples for 24 h. Subsequently, RAW 264.7 cells and the supernatant were collected for Elisa experiments.

### 2.4 Animal models and treatments

All animal experiments were performed according to the guidelines for laboratory animals established by Zhejiang University. During the study, rats were identified solely through labeled cage boxes without any direct markings on the animals themselves. Given the relatively large surgical wounds on the rats, each cage was housed with only one individual to minimize their distress and discomfort, as well as to prevent cross-inflicted wounds from mutual scratching that could exacerbate injuries. This arrangement was implemented to ensure the reliability and stability of experimental data to the greatest extent possible.

#### 2.4.1 Diabetic wound model

To create the type 1 diabetic model, Sprague Dawley rats (male, 6 weeks, 150–200 g) were injected with STZ (40 mg/kg) for 1 week. Rats with a blood glucose concentration of above 16.7 mM were considered as diabetic ones. Subsequently, full-layer skin wounds were established in all diabetic rats (approximately 10 mm in diameter) and grafted with saline, blank carbomer gel (KN), BDS and BDN. The wound dressing was changed every other day, digital images of wounds were acquired at different intervals (day 0, 7, 14, 21) and the wound area was calculated using ImageJ. The pathological status and healing process of wounds were evaluated by H&E staining and Masson’s trichrome staining. Keratin 10 expression was using immunohistochemical staining and CD31, *α*-SMA levels were determined using immunofluorescence staining. Western blot analysis was used to evaluate the anti-inflammatory activity and collagen synthesis of BDN: TNF-α, IL-10, TGF-*β*, IL-6, IL-1β, MMP-2/9, TIMP-1, *β*-actin. Skin tissues of rat wounds at day 21 were collected and used for bacterial culture by the standard plate counting assay to value the anti-bacterial activity of BDN.

#### 2.4.2 “Comb” burn model

The “comb” burn model was established following previous reports. (Fang et al., 2017). The burn wound area occupied approximately 4% of the total body surface area (TBSA). During the operation, the breathing and heart rates of the burned rats were carefully monitored to ensure that all of the rats were under anesthesia and pain-free before being allowed to recover from the anesthesia. Twenty animals were randomly assigned to four groups: Saline, KN, BDS and BDN group. The wounds were administered therapeutic agents at a dosage equivalent to 1 mg/cm^2^ of BDS. In the KN group, an equivalent volume blank hydrogel of BDN was applied. The wound sites were subsequently dressed with a four-layer petrolatum gauze as the primary (inner) dressing, overlaid with a four-layer cotton gauze as the secondary (outer) dressing. Following this, the wounds were secured using an elastic bandage, which was applied with gentle compression to ensure proper adherence and stabilization. Dressing changes were performed at 2-day intervals to maintain aseptic conditions and promote optimal wound healing dynamics.

The wound area was evaluated and recorded at different intervals (day 0, 4, 8, 14, 21). Skin samples were fixed in 4% paraformaldehyde, embedded in paraffin and sectioned at a thickness of 5 *μ*m using a rotary microtome. The pathological status and healing process of wounds were evaluated followed the detection in the diabetic wound model.

### 2.5 Safety evaluation

Various concentrations (4, 20, 100 *μ*g/mL) of BDS, and BDN were co-incubated with 2% of rabbit RBC for 1 h at 37°C, water was used as the positive control while saline was used as the negative control. After centrifuging at 3,000 rpm for 10 min, the supernatant was collected and the absolution was measured at 541 nm. The hemolysis ratio (HR) was calculated following the formula:

$$\left.\text{HR }\left(\%\right)=\left({\text{OD}}_{\text{sample}}-{\text{OD}}_{\text{negative}\;\text{contro}}l\right)\times 100\%\;/\;{\text{OD}}_{\text{positive}}-{\text{OD}}_{\text{negative}\;\text{control}}\right)$$

The liver and kidney were collected for conducting the histology analysis after the 21 days’ treatment was ended.

### 2.6 Statistical analysis

Data analysis was performed using GraphPad Prism 8.0.2 software to calculate mean ± standard de*via*tion (SD). Student’s t-test were used to assess significant differences between two groups, while One-way or Two-way ANOVA were used to assess significant differences when compared with more than two groups, while *p < 0.05, **p < 0.01, ***p < 0.001, ****p < 0.0001 indicating significance, and n s indicating no significance.

## 3 Results and discussion

### 3.1 Preparation and characterization of BDN hydrogel

As a macromolecular material, hydrogel can load drugs into a three-dimensional cross-linked network through physical or chemical methods. Compared with other commonly used topical dosage forms such as creams or petroleum jelly-based preparations, hydrogels offer the following advantages:

Firstly, the moist environment of hydrogels aligns better with the “moist wound healing theory” for wounds, demonstrating potential in accelerating granulation formation (Huang et al., 2024). In contrast, while petroleum jelly-based preparations can soften keratin, their poor air permeability may hinder the drainage of exudate, leading to wound maceration and anaerobic bacterial proliferation (Butko et al., 2021). The oil-water mixed system of creams is prone to loss with exudate (the water loss rate of cream bases is 3–5 times faster than that of gels), making it difficult to maintain a stable moist environment (Miastkowska et al., 2020). Secondly, Hydrogels possess strong swelling properties and the ability to absorb wound exudate (Tian et al., 2025). Although creams can promote drug penetration, their ability to absorb exudate is relatively poor, which may prolong healing time due to retained exudate. Thirdly, Hydrogels have a soft texture and are easy to remove, reducing mechanical damage and pain during dressing changes. Petroleum jelly gauze, on the other hand, tends to adhere to the wound when dry, and forced dressing changes may exacerbate injury (Wen and Shen, 2021). Fourthly, Hydrogels can optimize drug activity by adjusting pH, whereas creams or petroleum jelly-based preparations may limit drug effects due to the properties of their bases (Chen et al., 2023).

Considering the intrinsic properties of hydrogels, in addition to the bioactive drug substance-BDS, the prescription should also include gel base, humectant, preservative, and pH adjuster. Therefore, this study optimized the gel formulation using an orthogonal design to determine the optimal process for the preparation of BDN (Supplementary Table S1). As the preservative content in the gel is merely 0.1%, it has a negligible impact on the gel properties, hence preservative screening was not conducted.

Carbomer 940, sodium carboxymethyl cellulose (CMC-Na), and sodium alginate, as widely utilized gel bases, are recognized for their superior biocompatibility. Therefore, these three gel bases were given priority in the study. The experimental findings revealed that Carbomer 940 possesses superior gel-forming capabilities, enabling the formation of a uniform and refined gel at a 1% (w/v) concentration. This concentration yields a gel with moderate fluidity and viscosity, suitable as the gel base for this study. In contrast, the other gel bases, as depicted in Supplementary Figure S1, even at 10% sodium alginate and 5% CMC-Na, exhibit excessive fluidity, detrimental to drug adherence on wounds.

Humidity agents possess the capacity to adsorb and retain moisture, thereby preventing the evaporation of water from the skin surface, which in turn provides an extended humid environment for the skin or specific areas. In this study, the moisture retention of glycerol and 1,3-propanediol were evaluated. After the samples were incubated in an oven at 37°C for 48 h, the final moisturizing rate was 7.2% for the glycerol group and 5.5% for the 1,3-propanediol group. Additionally, the glycerol-enriched gel matrix was observed to be smoother, more refined, and less aerated. Hence, glycerol was selected as the preferred humectant in the BDN formulation.

**FIGURE 1:**
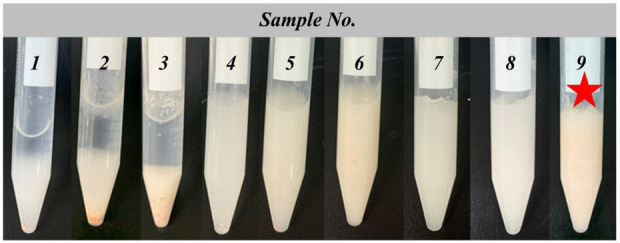
Appearances of the BDN hydrogel prepared with different compositions.

To optimize the recipe of BDN, we carried out an orthogonal optimization experiment in which the concentrations of Carbomer 940, BDS, pH value, and propylene glycol as investigation factors and the comprehensive sensory evaluation as an indicator (Supplementary Table S3). An orthogonal analysis was designed to identify factors that have a significant effect on the comprehensive sensory. The orthogonal design is listed in Supplementary Tables S1, S2, and the results revealed that sample 9 achieved the highest score, whereas samples 1–3 received the lowest scores (Figure 1). The range analysis (R) of the orthogonal test results indicated that the content of carbomer 940 had the greatest impact on the gel, followed by the amount of BDS, pH value, and glycerol content. The optimal formulation for BDN, denoted as A_3_B_3_C_2_D_1_, consisted of 5% carbomer 940, 2% BDS, pH adjusted to 7.40, and 5% glycerol.

Stability assessments demonstrated that the formulated gel maintained its integrity post-centrifugation, with no observable stratification or liquefaction, thereby confirming its favorable centrifugal stability (Supplementary Figure S2). After a 24-h period at both 60°C (Supplementary Figure S3) and −20°C (Supplementary Figure S4), the absence of precipitation, stratification was noted, thereby attesting to the gel’s exceptional thermostability across a broad temperature spectrum. The particle size distribution of the gel was analyzed in accordance with the methodology outlined in the Chinese Pharmacopoeia (2020 edition) under General Rule 0,982, First Method. Results showed that the prepared gel was both uniform and delicate, with the majority of particles measuring less than 10 *μ*m, significantly below the Pharmacopoeia’s stipulation of less than 180 *μ*m for gels in suspension form (Supplementary Figure S5).

**FIGURE 2:**
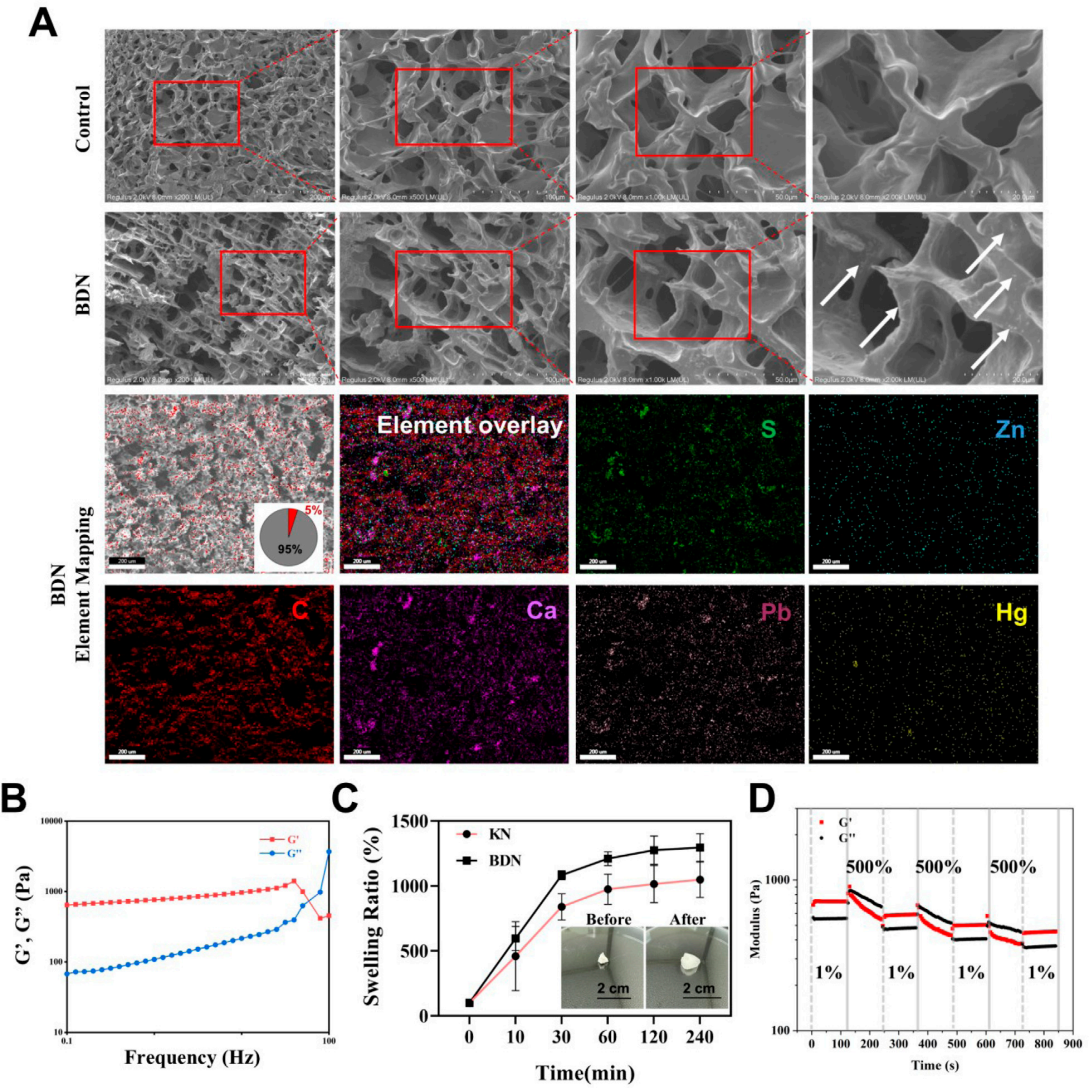
Characterization of BDN. **(A)** SEM images and element mapping of KN and BDN. **(B)** Storage modulus (G′) and loss modulus (G″) of BDN against frequency. **(C)** The swelling ratio (%) of BDN after being immersed in water at various times. **(D)** Thixotropic characterization of self-healing hydrogel at alternate strain of 1% and 500%, with angular frequency of 10 rad s^-1^.

Hydrogels are constituted by hydrophilic polymeric materials soluble in water, which undergo physical or chemical crosslinking to form a three-dimensional network-like structure. Macroscopically, the SEM images of BDN exhibit a more compact and ordered honeycomb-like network compared to the blank gel group. On a microscopic scale, the gel network of the blank gel group appears relatively smooth, whereas the BDN group displays numerous minute particles embedded within or on the surface of the gel network (Figure 2A). Combining the results of elemental mapping (Figure 2A) with prior slide analysis of the BDN gel (Supplementary Figure S5), suggests that these particles are likely to be microscopic granules of the BDS powder encapsulated within the gel. The elemental mapping further reveals a uniform distribution of representative elements from the powder within the hydrogel matrix, indicating a homogeneous dispersion of the powder without significant agglomeration. Further physicochemical characterization reveals that the storage modulus consistently exceeds the loss modulus at low frequencies, confirming the formation of an elastic hydrogel network in the BDN group (Figure 2B). Swelling analysis underscores the remarkable water-absorbing capacity of BDN, capable of absorbing approximately 1200% of its own weight in water (Figure 2C). The thixotropic properties of the hydrogel were analyzed with periodic strain changes (1% or 500% strain), which indicated that the hydrogel could recover its gel phase at 1% strain after gel rupture at 500% strain (Figure 2D). These results indicating that BDN exhibited self-healing property and recover to its original structure after broken. Collectively, these data underscore the exceptional physicochemical properties of the formulated BDN, rendering it a suitable candidate as a hydrogel dressing for wound care.

### 3.2 Cell behavior with BDN hydrogel

**FIGURE 3:**
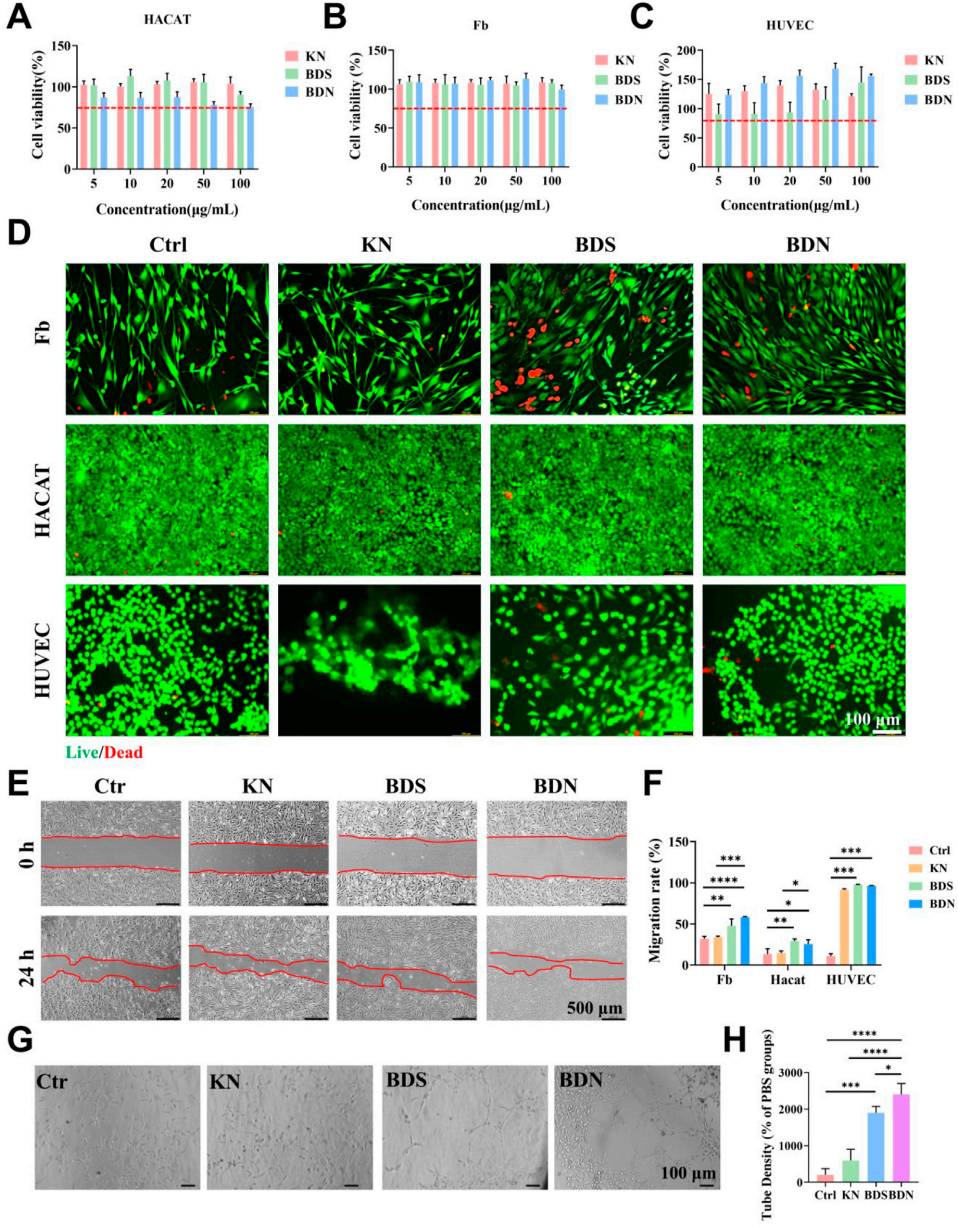
Cytotoxicity of KN, BDS and BDN against **(A)** Hacat, **(B)** Human Skin Fibroblasts (Fb) and **(C)** HUVEC cells (n = 6). The red dashed line represents a 75% survival rate. **(D)** Live/dead cell assay for cells treated with various samples. (Scale bar = 100 *μ*m, concentration of hydrogel extract = 100 *μ*g/mL). **(E)** BDN promoted the migration of Fb cells by cell scratch assay and **(F)** the quantity data of migration rate. Concentration of hydrogel extract was 100 *μ*g/mL, n = 3. **(G)** Representative images and **(H)** quantitative analysis of tube formation of HUVECs treated with different groups, scale bar = 100 *μ*m, n = 3. All data are presented as mean ± SD. Data were analyzed using contrast analyses following one-way ANOVA. *p < 0.05, **p < 0.01, ***p < 0.001, ****p < 0.0001. ns, No significant difference.

The wound healing process involves the participation of various cellular activities, such as the proliferation and migration of keratinocytes and fibroblasts, as well as the involvement of endothelial cells in the formation of new blood vessels. In this section, we focused on examining the effects of BDN on several representative cellular activities related to wound healing. The results of the CCK-8 assay (Figures 3A–C) combined with live/dead cell staining (Figure 3D) showed that BDN is highly biocompatible, as over 75% of Hacat and Fb cells remain viable even at concentrations up to 100 *μ*g/mL. Furthermore, it was observed that BDN and BDS exhibit a proliferation-promoting effect on HUVECs, consistent with literature reports that highlight the angiogenic activities of their primary constituents, calcined calamine and calcined plaster (Cheng et al., 2023). Scratch assays indicated that BDS and BDN significantly promoted the migration of the 3 cell lines, suggesting that BDS and BDN may accelerate wound healing by promoting cell migration (Figure 3E, F; Supplementary Figure S6). Tube formation assays showed that BDS and BDN significantly promoted the formation of new blood vessels, with BDN showing a stronger ability to promote angiogenesis than BDS (Figures 3G,H). The phenomenon attributed to the angiogenic potential of the carbomer gel itself, and on the other hand, it may be due to the gel’s certain degree of fibrinolytic effect on the red ochre, calcined calamine, calcined plaster contained in BDS (Cheng et al., 2023).

### 3.3 Antibacterial and anti-inflammation activity *in vitro*

Wound infection is one of the primary causes of delayed wound healing, particularly in diabetic patients where the hyperglycemic microenvironment of chronic wounds favors bacterial growth. Bacterial proliferation releases toxins and damages surrounding tissues, leading to necrosis that deprives nascent tissues of essential nutritional support on one hand, and exacerbating and perpetuating inflammatory responses on the other, thereby disrupting the normal wound healing process and delaying recovery (Yang et al., 2023; Chang and Nguyen, 2021; Uberoi et al., 2024).

**FIGURE 4:**
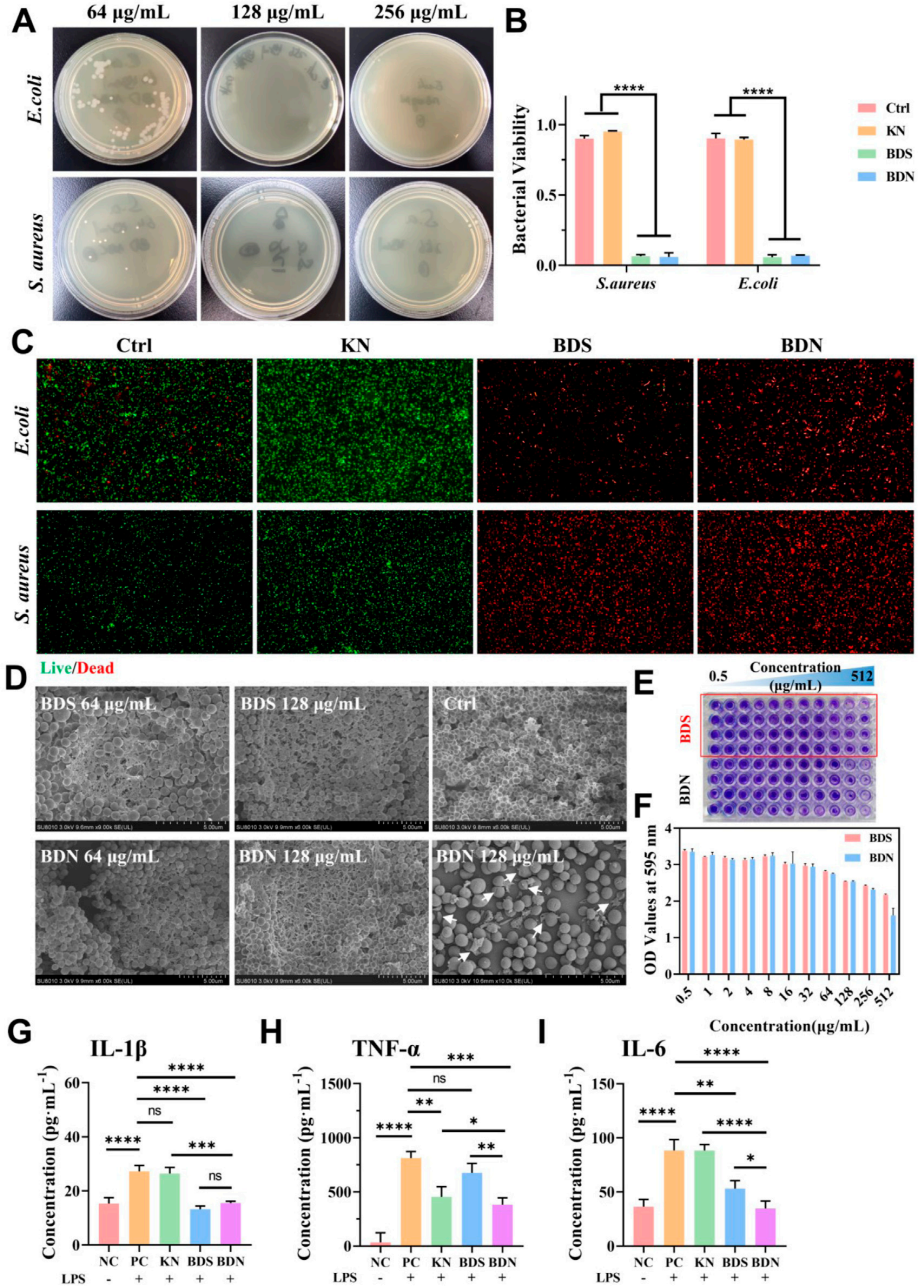
Evaluation of the antibacterial effect and antiinflammation of hydrogels. **(A)** Images of survival bacteria clones after the treatment of BDN hydrogels. **(B)** The quantitation data and **(C)** the live/dead staining of bacteria after the treatment of hydrogels, n = 3. (×40). **(D)** The corresponding SEM images of *Staphylococcus aureus* biofilm treated with different substances. Arrows pointed to the damaged bacterial structure. **(E)** The crystal violet staining results of the biofilm after being treated with various substances and **(F)** its OD values at 595 nm (n = 4). Expression levels of **(G)** IL-1β, **(H)** TNF-α, **(I)** IL-6 inflammatory factors in macrophages treated with various substances for 24 h, n = 3. All data are presented as mean ± SD. Data were analyzed using contrast analyses following one-way ANOVA. *p < 0.05, **p < 0.01, ***p < 0.001, ****p < 0.0001. ns, No significant difference.

The BDS and its components, such as red ochre and calomel, have been proven to possess broad-spectrum antibacterial properties (Cheng et al., 2023). In this section, the minimum inhibitory concentration (MIC) and minimum bactericidal concentration (MBC) of the prepared BDN were further measured by the microbroth dilution method. The results (Supplementary Figure S7) indicated that BDN significantly inhibited the growth of both *S. aureus* and *E. coli*, with an MIC value of 64 *μ*g/mL and an MBC value of 128 *μ*g/mL (Figure 4A). Since the BDS in BDN has poor solubility in water, the extraction of BDN used in this experiment was only co-incubated, diluted, shaken, and centrifuged with the medium. Therefore, considering the solubility factor, the actual MIC and MBC values of BDN should be lower. Live/dead bacterial staining (Figures 4B,C) further confirmed the comparable antibacterial capacity of BDN to BDS, demonstrating significant killing effects against both *S. aureus* and *E. coli*.

Bacterial biofilms are the major contributor to antibiotic resistance and recurrent wound infections. Firstly, biofilms impede the penetration of antibiotics or disinfectants, adsorb and inactivate certain antibiotics, thereby reducing the effective concentration of antibiotics within the biofilm. Simultaneously, the protective barrier of the biofilm allows bacteria time to adapt and develop antibiotic resistance. Furthermore, bacteria deep within biofilms often experience nutrient deprivation, leading to slow growth or even dormancy, enabling them to resist antibiotics targeting metabolic pathways through reduced metabolism. Therefore, disrupting bacterial biofilms is beneficial in reducing bacterial resistance to antimicrobials (Yang et al., 2023).

SEM (Figure 4D) revealed that most of the *S. aureus* cells in the control group were interconnected through a reticulated extracellular matrix (ECM) and forming large clusters, while the bacteria in BDS or BDN group were detached from the biofilm and exposed the ECM. Notably, more detached bacteria can be seen in the BDN group, indicating a stronger biofilm-disrupting ability than BDS. Additionally, SEM demonstrates that BDN significantly damages bacterial cell structures, with visible ruptures and efflux of cellular contents, suggesting that BDN exerts bactericidal effects by disrupting cell wall structures or causing imbalances in osmotic pressure across bacterial membranes. As the drug concentration in BDS and BDN groups decreases to 64 *μ*g/mL, the interlaced filamentous ECM structures transform into a buried-like structure while the bacterial density within the ECM increases significantly, suggesting reduced disruption of the bacterial biofilm structure occurred. The crystal violet staining results (Figures 4E,F) further confirmed that BDS and BDN can significantly disrupt *S. aureus* biofilms at a concentration of no lower than 128 *μ*g/mL.

Chronic inflammation is another significant factor contributing to delayed wound healing. In this study, an *in vitro* inflammatory model was constructed using LPS-induced macrophage polarization, and the anti-inflammatory abilities of BDN and BDS were evaluated by measuring the changes of inflammatory factor levels. The results showed that macrophages pretreated with BDN or BDS significantly alleviated or even reversed the release of various inflammatory factors, such as IL-1β, IL-6, and TNF-α (Figures 4G–I). Furthermore, BDN demonstrated stronger inhibitory effects on IL-6 and TNF-α compared to BDS, which may be partly attributed to the anti-inflammatory properties of carbomer gel itself and the interaction between carbomer and BDS (Cheng et al., 2023).

### 3.4 Therapeutic effects on the diabetic chronic wound healing

**FIGURE 5:**
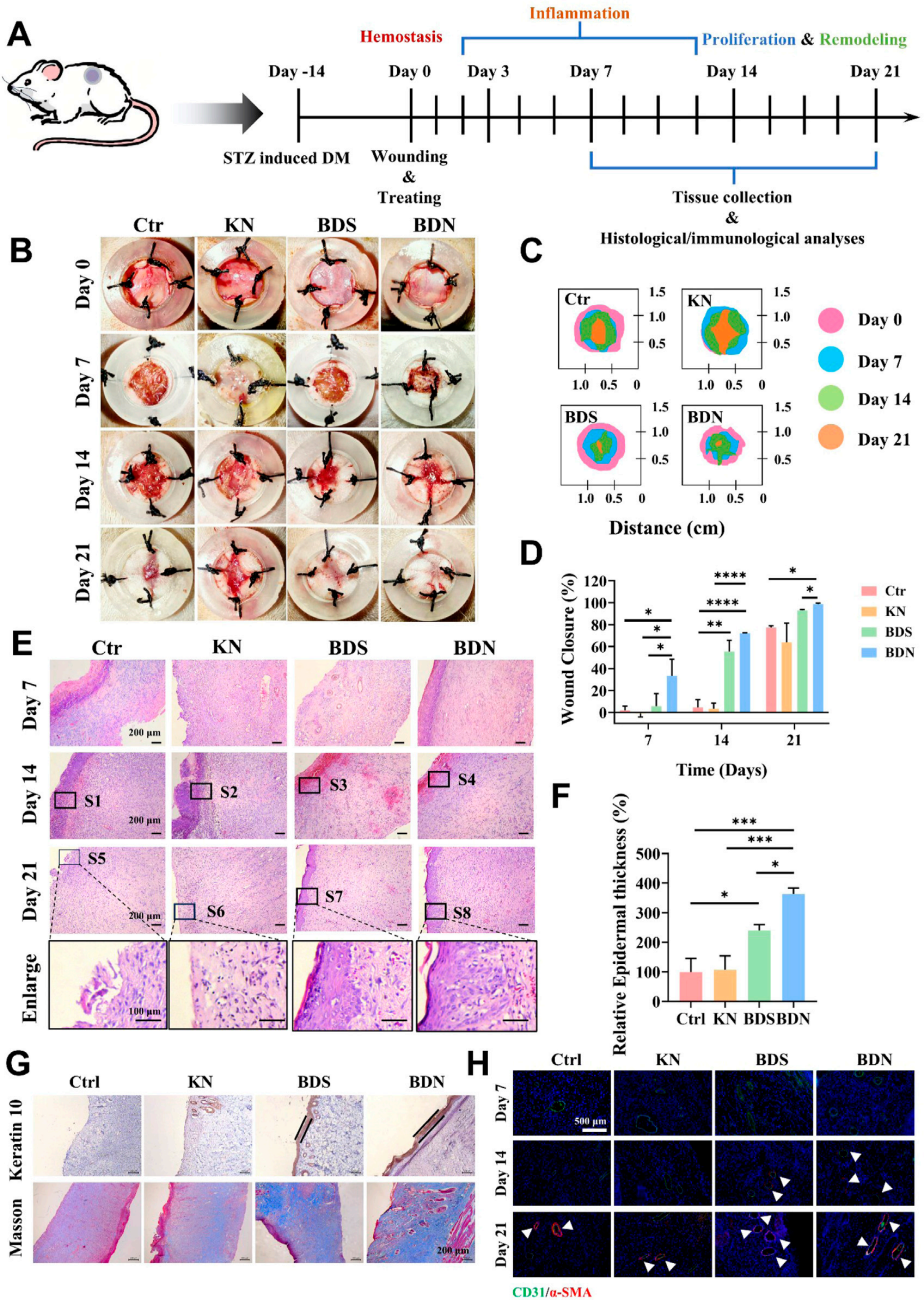
The efficiency of the BDN hydrogel on a full-thickness wound healing of diabetic rat. **(A)** Schematic representation of *in vivo* wound healing experiment process. **(B)** Photographs of wounds treated with different samples on day 0, 7, 14, 21. **(C)** Traces of wound closure over 21 days and **(D)** the Quantification of the wound healing rate (n = 3). **(E)** H&E images of wound tissue with varied treatments at different timepoint. **(F)** Quantitative analysis of epidermal thickness. **(G)** Immunohistochemical results of keratin 10 and Masson’s trichrome staining of wound tissue with different treatments on day 21, n = 3. **(H)** *α*-SMA and CD31 immunofluorescence staining for each group at different time. All data are presented as mean ± SD. Data were analyzed using contrast analyses following one-way or two-way ANOVA. *p < 0.05, **p < 0.01, ***p < 0.001, ****p < 0.0001. ns, No significant difference.

The favorable biocompatibility, remarkable antibacterial, anti-inflammatory, and angiogenesis-promoting properties exhibited by BDN *in vitro* have prompted us to further validate its ability to facilitate diabetic chronic wound healing *in vivo*. In this study, a full-thickness diabetic rat wound model was established (Figure 5A). Our findings revealed that both BDN and BDS significantly accelerated the wound healing process, with BDN demonstrating superior wound healing speed and quality compared to BDS. Gross observation revealed that the wound healing rate in the BDN group approached 40% on day 7 post-treatment, whereas it was approximately 20% in the BDS group. In contrast, the wound sizes in the control and KN groups remained virtually unchanged, and even slightly increased due to infection-induced necrotic, indirectly confirming the antibacterial properties of BDS and BDN (Figures 5B–D). On Day 14, significant inflammatory cell recruitment was observed in the Ctrl and KN groups, correlating with visible bacterial infections (Figure 5E). The occasional erythrocytes in the BDS and BDN groups may result from minor scab dislodgment during wound debridement before sample collection, potentially causing slight bleeding (Figure 5E; Supplementary Figure S8). Additionally, combined with K10 staining (Figure 5G), the results showed that the newly formed epidermis was significantly thicker in the BDS and BDN groups compared to the control and KN groups (Figure 5F), with a tighter attachment to the subcutaneous tissue and a more abundant stratum spinosum in the BDN group, indicating higher healing quality. Masson staining (Figure 5G) showed that both BDN and BDS promoted collagen deposition, with BDN exhibiting a more compact and uniform distribution of collagen fibers in a basketweave pattern within the dermis. CD31 and *α*-SMA were used to label vascular endothelial cells and smooth muscle cells, respectively. The immunofluorescence staining results revealed that the presence of mature vessels in the wounds of the BDS and BDN groups at an earlier stage (day 14), with a higher proportion of mature vessels in the BDN group. Additionally, on day 21, the density of mature vessels in the BDS and BDN groups was significantly higher than that in the control and KN groups, indicating the angiogenesis-promoting ability of BDS and BDN *in vivo* (Figure 5H; Supplementary Figure S10).

**FIGURE 6:**
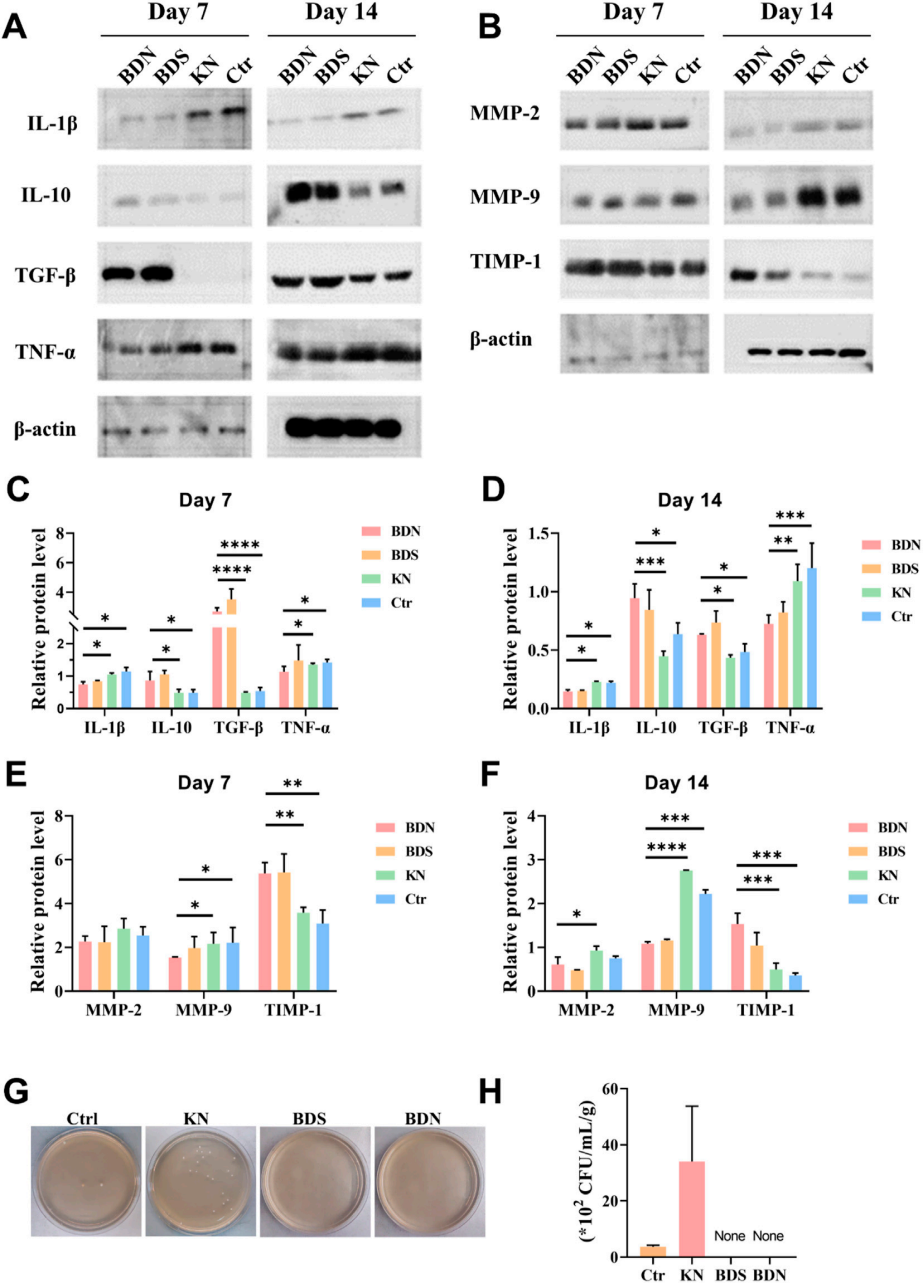
The representative protein levels related to inflammation or collagen formation. The Western blot results of **(A)** inflammation factors and **(B)** factors related to collagen formation in wounds. The quantitative results of **(C,D)** inflammation factors and **(E,F)** factors related to collagen formation, n = 3. **(G)** Representative photographs and **(H)** quantitative data of bacterial culture from the skin tissue of rat wounds at day 21 characterized by the standard plate counting assay (n = 3). All data are presented as mean ± SD. Data were analyzed using contrast analyses following one-way or two-way ANOVA. *p < 0.05, **p < 0.01, ***p < 0.001, ****p < 0.0001. ns, No significant difference.

Inflammation and proteolysis are pivotal in the orchestration of tissue regenerS7ation and wound repair. To elucidate the impact of BDS and BDN on these processes, we quantified inflammatory mediators and factors associated with protein breakdown in regenerative tissues using Western blot. Our findings indicate that both BDS and BDN markedly attenuated the expression of pro-inflammatory cytokines, including IL-1β and TNF-α, while concurrently augmenting the levels of anti-inflammatory mediators, IL-10 and TGF-*β*. This modulation was sustained over a 14-day period, facilitating a seamless progression of the wound healing cascade from the inflammatory to the proliferative and remodeling phases (Figures 6A,C,D).

Matrix metalloproteinases (MMPs) and tissue inhibitors of metalloproteinases (TIMPs) play crucial roles in regulating the metabolic balance between ECM synthesis and degradation, which is closely related to wound healing and tissue remodeling. Comparative analysis with control and KN groups revealed that BDS and BDN significantly curtailed the wound tissue expression levels of MMP-2/9 at multiple time points post-treatment, and concurrently elevated TIMP-1 content. These results suggest a superior capacity of BDS and BDN to enhance tissue protein synthesis compared to the control and KN groups (Figures 6B,E,F).

Finally, bacterial cultures were performed on homogenized wound tissues from each group. The results showed that the bacterial growth was observed in both the control and KN groups, whereas BDS and BDN groups exhibited significant antibacterial effects, consistent with the results of *in vitro* antibacterial experiments (Figures 6B,E). Notably, the KN group exhibited a higher bacterial load and more severe infection in wound tissues compared to the control group, which explained the slowest wound healing rate and higher MMP-2/9 content related to protein degradation in the KN group (Figures 6G,H). These observations underscore the propensity for bacterial proliferation in moist environments and highlight the critical role of effective antibacterial strategies in wound management.

In conclusion, the aforementioned series of results indicate that both BDS and BDN possess the ability to accelerate chronic wound healing. However, BDN exhibits superior healing speed and quality compared to BDS, which may be attributed to the synergistic healing effect between BDS and the moist environment provided by the hydrogel.

### 3.5 *In vivo* healing performance of the BDN hydrogel in a “comb” burn model

**FIGURE 7:**
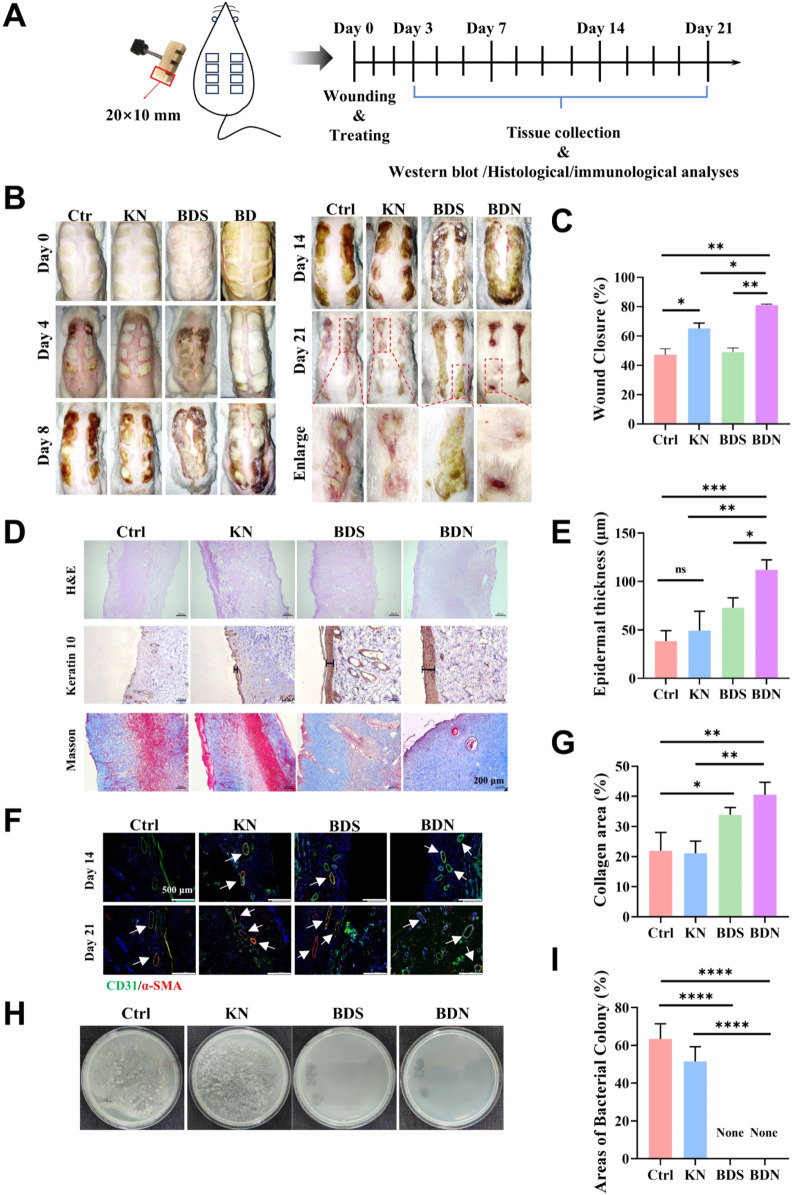
*In vivo* healing performance of the BDN hydrogel in a “comb” burn model. **(A)** Schematic illustration of the experimental procedure for treating deep burn wounds. **(B)** Photographs of wounds on day 0, 4, 8, 14, 21 from the different treatments. **(C)** Wound closure rate measured by ImageJ (n = 3). **(D)** H&E, keratin 10 and masson staining in different treatment groups. The thickness of epidermal **(E)** and **(G)** collagen areas in different treatment groups (n = 3). **(F)** *α*-SMA and CD31 immunofluorescence staining for each group at different time. **(H)** Representative photographs and **(I)** quantitative data of bacterial culture from the skin tissue of rat wounds at day 21 characterized by the standard plate counting assay (n = 3). All data are presented as mean ± SD. Data were analyzed using contrast analyses following one-way or two-way ANOVA. *p < 0.05, **p < 0.01, ***p < 0.001, ****p < 0.0001. ns, No significant difference.

Burns and scalds, frequent occurrences in everyday life, demand swift primary care guided by the quintessential “rinse, remove, soak, cover, and transport” approach. Concurrently, the judicious and timely use of pharmaceuticals is imperative to prevent the escalation of wound severity, avert infection, and preclude critical sequelae such as shock and sepsis. Given the remarkable antibacterial and anti-inflammatory properties of BDS and BDN previously demonstrated in chronic wounds, this section we have utilized a high-temperature brass comb to induce a deep second-degree burn model in rats and following evaluated the wound-healing effects of BDN on deep second-degree burns. A rigorous 21-day monitoring of dynamic wound progression in rats were conducted (Figures 7A,B). As depicted in Supplementary Figure S9, the successful establishment of the deep second-degree burn model was primarily determined by the histological examination. H&E staining indicated full-thickness epidermal necrosis and superficial dermal coagulation necrosis in rats. While some follicular necrosis with cystic cavitation was present, residual skin appendages remained, distinguishing these burns from third-degree burns and aligning with the characteristics of deep second-degree burns. Necrosis emerged on day 4 within the untreated control and BDS groups, while their onset was deferred to day 8 in the KN and BDN groups, implicating that the moist environment provided by the hydrogel could postpone skin tissue necrosis in the early stages of burns. Notably, the majority of the BDN group remained non-necrotic until day 14, underscoring a synergistic effect between the gel matrix and BDS contained in BDN in preventing tissue necrosis. From day 8 to day 14, the dense eschar formed by skin necrosis and coagulation, conspicuously impeded transdermal drug permeation, particularly pronounced in the BDS and control groups. On day 21, pronounced bleeding was observed upon trimming the eschar at the edges of the BDN group’s wounds, indicating that BDN could improve wound microcirculation and potentially promote angiogenesis, which was further corroborated by immunofluorescence analysis for neovascularization (Figure 7F). Conversely, significant infection and swelling were observed in the untreated control and KN groups, with the former exhibiting subcutaneous infection. Notably, the wound area in the BDN group was significantly smaller than the other three groups, and no infection occurred, demonstrating that BDN significantly accelerated healing in acute deep second-degree burn wounds (Figure 7C).

H&E staining results showed that the newly formed epidermis in the BDN group was significantly thicker than that in the other groups. Besides, BDS alone also had a certain pro-re-epithelialization effect (Figures 7D,E). Further, K10 immunostaining elucidated that BDN significantly bolstered keratinocyte differentiation and fortified the keratinocyte barrier function, surpassing BDS and KN (Figure 7D). Moreover, BDN prominently facilitated collagen deposition, with BDS also demonstrating a notable contribution in this regard (Figures 7D,G). The results of bacterial culture of wound tissues were consistent with visual observations, with obvious infections in the untreated control and KN groups, while BDS and BDN exhibited significant antibacterial effects (Figures 7H,I).

Collectively, these findings confirmed that BDN accelerates deep second-degree burn wound healing through moisture retention, antibacterial activity, promotion of wound re-epithelialization, angiogenesis, and keratinocyte differentiation.

In clinical practice, it has been observed that deep burn wounds are not usually stable and undergo a dynamic progress that results in the deepening and expansion of the initial burn area, a process defined as burn-wound progression (Fang et al., 2017). This progressive damage can induce the conversion of the burn wound from a superficial partial-thickness burn to a deep partial-thickness or full-thickness burn and the development of initially unburned skin into part of the burn wound, which will affect both the appearance and function of the affected area, imposing significant treatment and psychological burdens on patients. Researches have shown that the inflammatory cascade response following burns results in excessive release of inflammatory factors, triggering tissue edema and ischemia, which may be the primary cause of progressive deepening in burn wounds (Fang et al., 2017). Therefore, controlling the inflammatory cascade response in burn wounds is crucial for preventing their progressive deepening.

**FIGURE 8:**
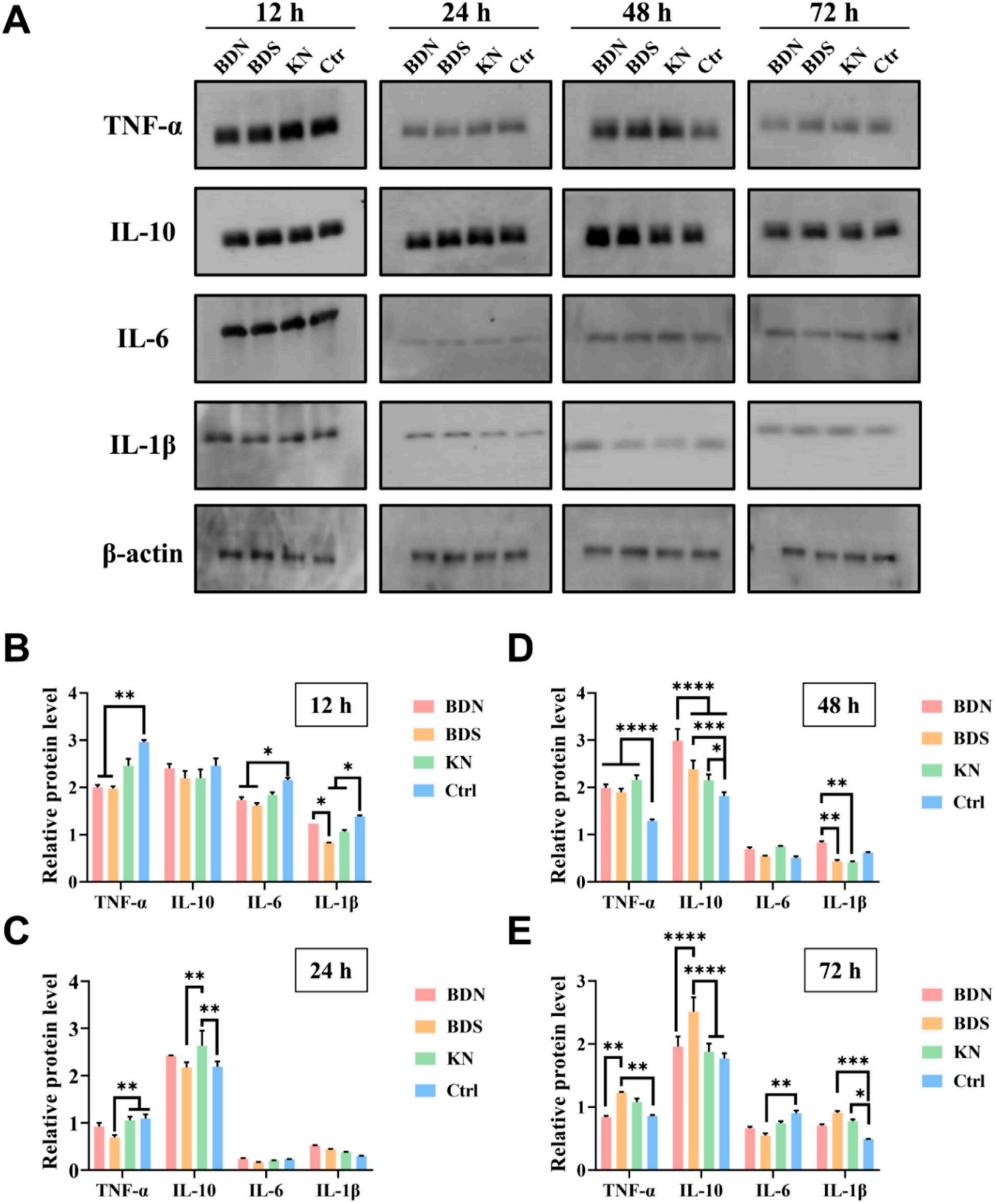
The **(A)** Western blot results and relative protein levels of inflammation factors measured from the skin tissue of rat wounds at **(B)** 12 h, **(C)** 24 h, **(D)** 48 h and **(E)** 72 h. All data are presented as mean ± SD. Data were analyzed using contrast analyses following one-way or two-way ANOVA. *p < 0.05, **p < 0.01, ***p < 0.001, ****p < 0.0001. ns, No significant difference.

Given that progressive wound deepening typically occurs within 72 h and directly correlates with excessive inflammatory cascades at the burn site, hence, we focused on examining the levels of inflammatory factors in various groups after intervention within 72 h post-burn (Figure 8A). Both BDS and BDN exerted rapid anti-inflammatory effects by reducing the levels of TNF-α and IL-6 after 12 h of administration (Figures 8A,B). Notably, BDS, due to its direct contact with the wound, was able to decrease IL-1β levels faster than BDN. Moreover, the use of KN for moisturizing within 48 h post-burn was found to elevate IL-10 levels in the wound, aiding in suppressing the inflammatory storm and preventing wound necrosis and progressive deepening (Figures 8C,D). After 48 h post-burn, compensatory self-regulation occurred in response to the earlier inhibition of TNF-α by BDS, BDN, and KN, leading to an upregulation of TNF-α levels and a significant upregulation of the anti-inflammatory factor IL-10 (Figure 8D). Notably, the IL-10 levels in the BDN group were significantly higher than those in the other three groups. At 72 h, TNF-α, IL-6, IL-1β, and IL-10 levels in the BDN group were the first to decrease and reach a balanced state (Figure 8E). These data indicate that BDN was able to more rapidly and smoothly guide burn wounds through the inflammatory storm, preventing their progressive deepening, which is consistent with the overall wound observations.

During the pathological progression of burns, macrophage polarization status undergoes dynamic evolution, generally manifesting an initial pro-inflammatory M1 phenotype post-burn, which ultimately transitions to an anti-inflammatory M2 phenotype (Zhu Z. et al., 2023). Our results revealed significantly higher M2 macrophage infiltration in the BDN group compared to the other three groups, with the BDS group showing moderate levels, and the KN group marginally higher than control (Supplementary Figure S11). These findings suggest that M2 macrophages in the BDN group exert enhanced anti-inflammatory effects within 72 h, facilitating a rapid transition of the wound environment through the inflammatory storm. This early modulation of inflammation by M2 macrophages in the BDN group also explains the subsequent superior angiogenesis and collagen deposition observed during the wound healing cascade in this cohort.

### 3.6 Safety evaluation

Safety is one of the most crucial evaluation metrics for drugs used in *in vivo* therapy. The results demonstrate that neither BDS nor BDN poses a risk of hemolysis (Supplementary Figure S12A; Supplementary Figure S12B). Kidney is one of the primary target organs for the mercury toxicity of BDS (Yanli et al., 2012), and H&E staining revealed significant dilation of renal tubules in the BDS group after 4 weeks of use, whereas this symptom was markedly alleviated in the BDN group (Supplementary Figure S12C). The formulation of BDS comprises heavy metals, including lead and mercury, which are considered essential medicinal components and constitute a distinctive characteristic of this prescription (Yanli et al., 2012; Lu et al., 2012). Existing literature indicates that the toxicity of BDS is predominantly associated with its mercury and lead content, with mercury being the principal contributor and lead serving as a secondary factor (Yanli et al., 2012). The primary organ affected by its toxicity is the kidney. Presently, there is a paucity of clinical reports addressing dermatological toxicity or allergic reactions following the administration of BDS. Consequently, this study concentrated on examining its renal toxicity. Notably, within the formulation of BDS, components such as calcined os draconis, calcined gypsum, and calcined calamine exhibit certain detoxifying properties. Specifically, calcined calamine has been shown to mitigate the mercury-induced toxicity of BDS. Zinc ions, as the main component of calcined calamine, play a role in reducing mercury-induced renal toxicity through various mechanisms, such as competitively inhibiting the binding of mercury to plasma proteins, thereby decreasing renal accumulation (Lu et al., 2011; He et al., 2012). In comparison to BDS, the renal toxicity associated with BDN was further alleviated in this study. It is hypothesized that this effect is primarily due to BDN’s enhanced dissolution of zinc ions present in BDS, which subsequently amplifies the detoxification efficacy of calcined calamine on mercury ions.

Additionally, no organic damage to the liver was detected in any of the groups (Supplementary Figure S12C). Consequently, the BDN prepared in this study not only exhibits excellent wound healing capabilities but also possesses good biosafety, mitigating renal damage associated with the long-term use of BDS.

## 4 Conclusion

In this study, we have reformulated and optimized BDS from a powder to BDN. Its efficacy in promoting the healing of diabetic chronic wounds and acute second-degree burn wounds also be investigated. Ultimately, we found that there exists a synergistic effect between carbomer gel and BDS. The prepared BDN not only retains BDS’s abilities in promoting cell migration, stimulating angiogenesis, exerting antibacterial and anti-inflammatory effects but also, due to the moisturizing properties of the hydrogel and its potential solubilizing capacity for BDS, further enhances the wound healing speed, quality, and safety. The study not only expands the clinical application scope of BDS, providing a novel tool for clinical acute and chronic wound care but also offers a general approach for the modification of powder dosage forms.

## Data Availability

The original contributions presented in the study are included in the article/Supplementary Material, further inquiries can be directed to the corresponding authors.
