# Supplementary material for: BaDuShengJi hydrogel accelerates diabetic wounds healing and regeneration of deep burn injury by anti-bacteria, anti-inflammation and promoting epithelialization
Source: Front Pharmacol. 2025 Jun 4;16:1580994. doi: 10.3389/fphar.2025.1580994 (PMC12174157; doi:10.3389/fphar.2025.1580994)
Supplement: Supplementary file 1 [file Supplementaryfile1.docx]

**Supporting Information**

**BaDuShengJi Hydrogel Accelerates Diabetic Wounds Healing and Regeneration of Deep Burn Injury by Anti-bacteria, Anti-inflammation and Promoting Epithelialization**

*Yuxiang Zhang^1, #^, Yuanyuan Yu^2, 3, #^, Lien Yu^1^, Fang Wang^2, 3^, Fuwei Yang^2, 3^, Shiyu Liang^4^, Wen Xu^5^, Mengying Ji^5^, Yinuo Geng^6^, Jingwei Xue^2, *^, Chunmao Han^1, *^, Zhongtao Zhang^1, 2, *^, Yilin Zhang^7 ,8, 9, *^*

^1^ Department of Burns and Wound Care Center, Second Affiliated Hospital, College of Medicine, Zhejiang University, Hangzhou, 310009, China

^2^ Tumor Precise Intervention and Translational Medicine Laboratory, The Affiliated Taian City Central Hospital of Qingdao University, Taian, 271000, China

^3^ Shandong Provincial Key Medical and Health Laboratory of Microenvironment-Responsive Biomedical Materials, Taian City Central Hospital, Taian, 271000, China

^4^ Department of Nursing Division, First Affiliated Hospital, College of Medicine, Zhejiang University, Hangzhou, 310006, China

^5^ Department of Nursing Division, Second Affiliated Hospital, College of Medicine, Zhejiang University, Hangzhou, 310009, China

^6^ Department of Nursing, Taishan Vocational College of Nursing, Taian, 271000, China

^7^ Taishan Academy of Medical Sciences, The affiliated Taian City Central Hospital of Qingdao University, Taian, 271000, China

^8^ Postdoctoral Mobile Station, Shandong University of Traditional Chinese Medicine, Jinan, 250355, China

^9^ Affiliated Hospital of Shandong Institute of Traditional Chinese Medicine, Jinan, 250014, China

^#^ Yuxiang Zhang and Yuanyuan Yu made equal contributions to this work.

* Corresponding author (E-mail: zhangzhongtao@qdu.edu.cn; Tel: +86 05386298085)

**Table of Contents**

| **NO.** | **Content** | **Page#** |
| --- | --- | --- |
| 1 | Materials | S3 |
| 2 | Evaluation the biocompatibility of BDN *in vitro* | S3 |
| 3 | *In vitro* cell migration evaluation of BDS and BDN | S3 |
| 4 | Tube formation assay | S3 |
| 5 | Table S1 | S4 |
| 6 | Table S2 | S4 |
| 7 | Table S3 | S5 |
| 8 | Figure S1 | S6 |
| 9 | Figure S2 | S6 |
| 10 | Figure S3 | S6 |
| 11 | Figure S4 | S6 |
| 12 | Figure S5 | S7 |
| 13 | Figure S6 | S7 |
| 14 | Figure S7 | S7 |
| 15 | Figure S8 | S8 |
| 16 | Figure S9 | S8 |
| 17 | Figure S10 | S8 |
| 18 | Figure S11 | S9 |
| 19 | Figure S12 | S9 |

**Materials**

Carbomer 940, glycerol, sodium alginate, triethanolamine, 1,3-Propanediol, carboxymethyl cellulose were purchased from Macklin Biochemical Technology Co., Ltd., BDS was obtained from Jianmin Pharmaceutical Group Co., Ltd. The antibodies were purchased from Proteintech, China. Hacat, Human Skin Fibroblasts (Fb), HUVEC cells were obtained from ATCC (American Type Culture Collection) and cultured in Dulbecco's Modified Eagle's Medium (DMEM) supplemented with 10% (v/v) FBS under standard conditions (humidified atmosphere containing 95% air and 5% CO_2_, 37 °C). ELISA kits of IL-1*β*, TNF-*α*, IL-6 were purchased from Beijing Biosynthesis Biotechnology Co., Ltd.

**Evaluation the biocompatibility of BDN *in vitro***

Cytotoxicity assessment of BDS and BDN were conducted using CCK-8 assay and live/dead cell staining. For the CCK-8 assay: freeze-dried hydrogel was soaked in DMEM medium for 24 h to obtained hydrogel extract at a concentration of 100 *μ*g/mL. Hacat, Fb and HUVEC cells were seeded in 96-well plates at a density of 5×10^3^ cells per well and cultured in DMEM medium with 10% FBS. After 24 h, the medium was replaced with the hydrogel extract and co-cultured with cells for 24 h. CCK-8 solution was added to each well and incubated for 2 h, followed by measuring the absorbance at 450 nm. For live/dead cell staining, staining reagent was added to each well and incubated for 20 min at 37℃. After washing with PBS for twice, live and dead cells were observed by the fluorescence microscope.

***In vitro* cell migration evaluation of BDS and BDN**

Hacat, Fb, HUVEC cells were seeded in 6-well plates until fully confluent. Scratches were made using a pipette tip. Cells were cultured for 24 h in DMEM with KN, BDS, or BDN extracts to a final concentration of 100 *μ*g/mL. Images were captured at 24 h using an inverted microscope.

**Tube formation assay**

Thaw Matrigel™ overnight in an ice bath and precool pipette tips and 96-well plates at 4°C. Mix Matrigel™ with FBS-free DMEM at 1:1 ratio and add 50 *µ*L to each well of pre-cooled plates. Incubate at 37°C for 30 minutes to form a gel. Seed 1×10^4^ HUVECs per well in FBS-free medium onto the Matrigel™-coated plates. Add KN, BDS, and BDN extracts to a final concentration of 100 *μ*g/mL. Incubate for 24 h at 37°C. Capture images using a brightfield microscope. Analyze tube formation using Image J software. PBS serves as the control group.

**Table S1** Levels and factors of the orthogonal experimental design for BDN hydrogel preparation

| Levels | Factors | | | |
| --- | --- | --- | --- | --- |
|  | A | B | C | D |
|  | Carbomer 940  （%） | BDS  （%） | pH | Glycerol  （%） |
| 1 | 1 | 0.5 | 7.40 | 2 |
| 2 | 3 | 1 | 6.50 | 5 |
| 3 | 5 | 2 | 6.00 | 10 |

**Table S2** Combination of variables of the orthogonal experimental design for BDN hydrogel preparation .[ 9 runs (3 levels and 4 factors)]

| Test number | Factor | | | | Residual | Uniformity | Spread ability | Adhesive  -ness | Centrifuge stability | Score |
| --- | --- | --- | --- | --- | --- | --- | --- | --- | --- | --- |
|  | A | B | C | D |  |  |  |  |  |  |
| 1 | 1 | 1 | 1 | 1 | 0 | 0 | 0 | 0 | 0 | 0 |
| 2 | 1 | 2 | 2 | 2 | 0 | 0 | 0 | 0 | 0 | 0 |
| 3 | 1 | 3 | 3 | 3 | 0 | 0 | 0 | 0 | 0 | 0 |
| 4 | 2 | 1 | 2 | 3 | 15 | 5 | 15 | 10 | 20 | 65 |
| 5 | 2 | 2 | 3 | 1 | 15 | 10 | 15 | 10 | 20 | 70 |
| 6 | 2 | 3 | 1 | 2 | 15 | 25 | 20 | 20 | 20 | 100 |
| 7 | 3 | 1 | 3 | 2 | 25 | 10 | 20 | 15 | 25 | 95 |
| 8 | 3 | 2 | 1 | 3 | 25 | 10 | 20 | 25 | 25 | 105 |
| 9 | 3 | 3 | 2 | 1 | 25 | 15 | 20 | 25 | 25 | 110 |
| K1 | 0 | 53.3 | 68.3 | 58.3 |  |  |  |  |  |  |
| K2 | 78.3 | 58.3 | 56.7 | 65.0 |  |  |  |  |  |  |
| K3 | 103.3 | 68.3 | 55.0 | 56.7 |  |  |  |  |  |  |
| R | 103.3 | 15.0 | 13.3 | 8.3 |  |  |  |  |  |  |
| Proportion | A>B>C>D | | Best | A_3_B_3_C_2_D_1_ | |  |  |  |  |  |

**Table S3** Comprehensive sensory evaluation method for optimizing BDN

| **Evaluation Criteria** | **Score Range** | **Scoring Method** |
| --- | --- | --- |
| Residue | 0-25 | 0: Fluid-like, unable to effectively retain on the skin;  Divided into 5 levels based on fluidity: 0, 5, 10, 15, 20, 25;  25: Gel-like consistency, does not slip off the skin surface. |
| Uniformity | 0-25 | 0: Obvious depressions or protrusions on the gel surface, significant granular feeling or visible clumps on the gel surface;  Divided into 5 levels based on the uniformity of the gel surface: 0, 5, 10, 15, 20, 25;  25: Smooth gel surface, no granular feeling, uniform color. |
| Spreadability | 0-25 | 0: Large-area gel spillage or inability to spread;  Divided into 5 levels based on spreadability: 0, 5, 10, 15, 20, 25;  25: Smooth spreading without interruption. |
| Adhesiveness | 0-25 | 0: Poor adhesion, falls off multiple times;  Divided into 5 levels based on the degree of adhesion: 0, 5, 10, 15, 20, 25;  25: Apply gel to the knuckle, perform 10 flexion and extension movements, gel remains tightly adhered to the skin without falling off. |
| Centrifugal Stability | 0-25 | 0: Severe stratification;  Divided into 5 levels based on the degree of centrifugal stability: 0, 5, 10, 15, 20, 25;  25: No stratification, uniform drug dispersion; |


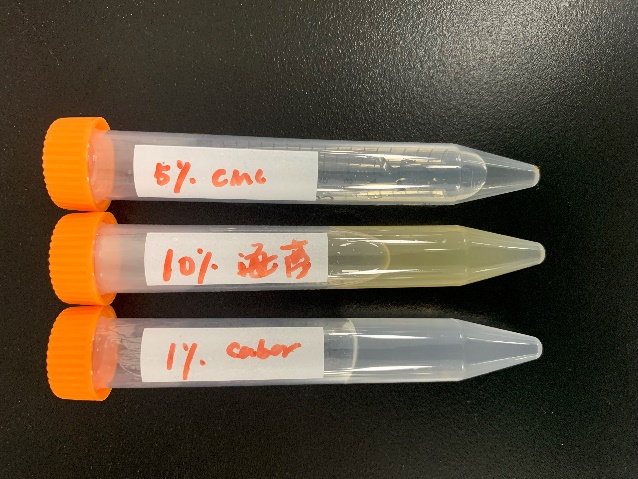


**Figure S1.** The state of different gel matrices in horizontal state.（Left to right：1% Carbomer 940, 10% Sodium Alginate, 5% CMC-Na）


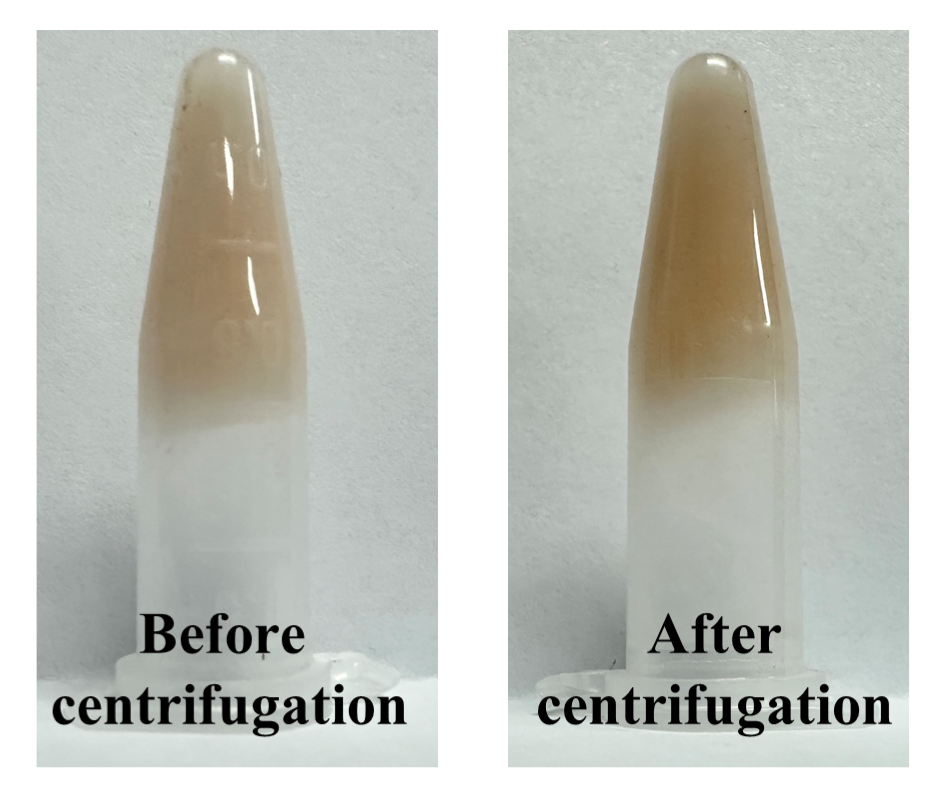


**Figure S2.** The state changes of BDN before and after centrifugation.


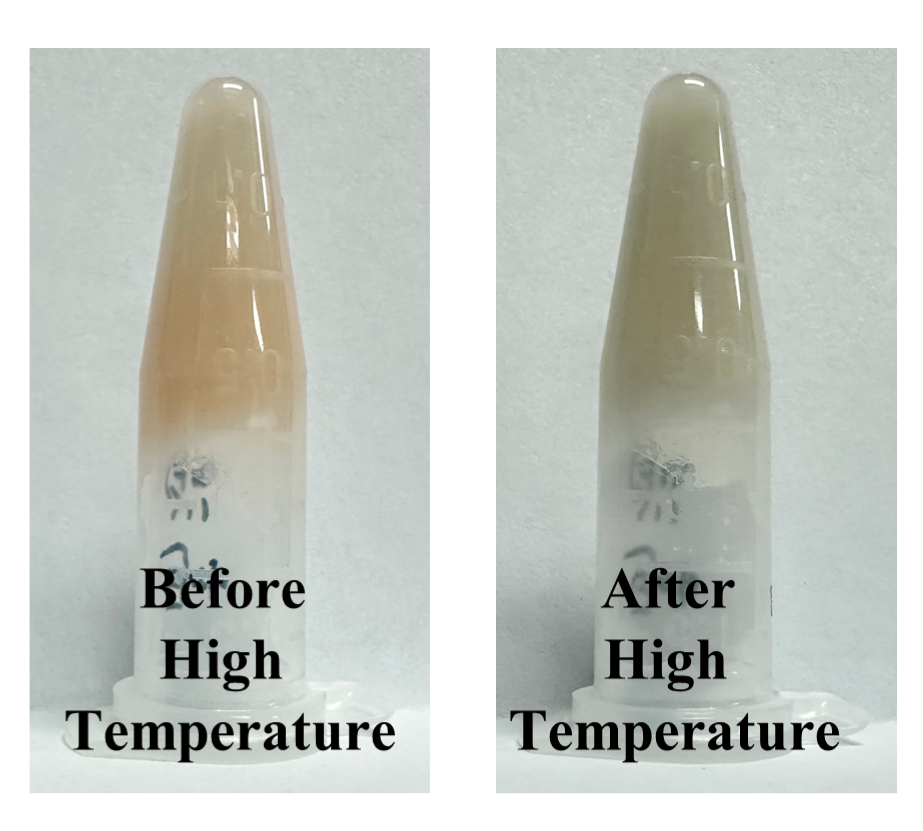


**Figure S3.** The state changes of BDN before and after high temperature.


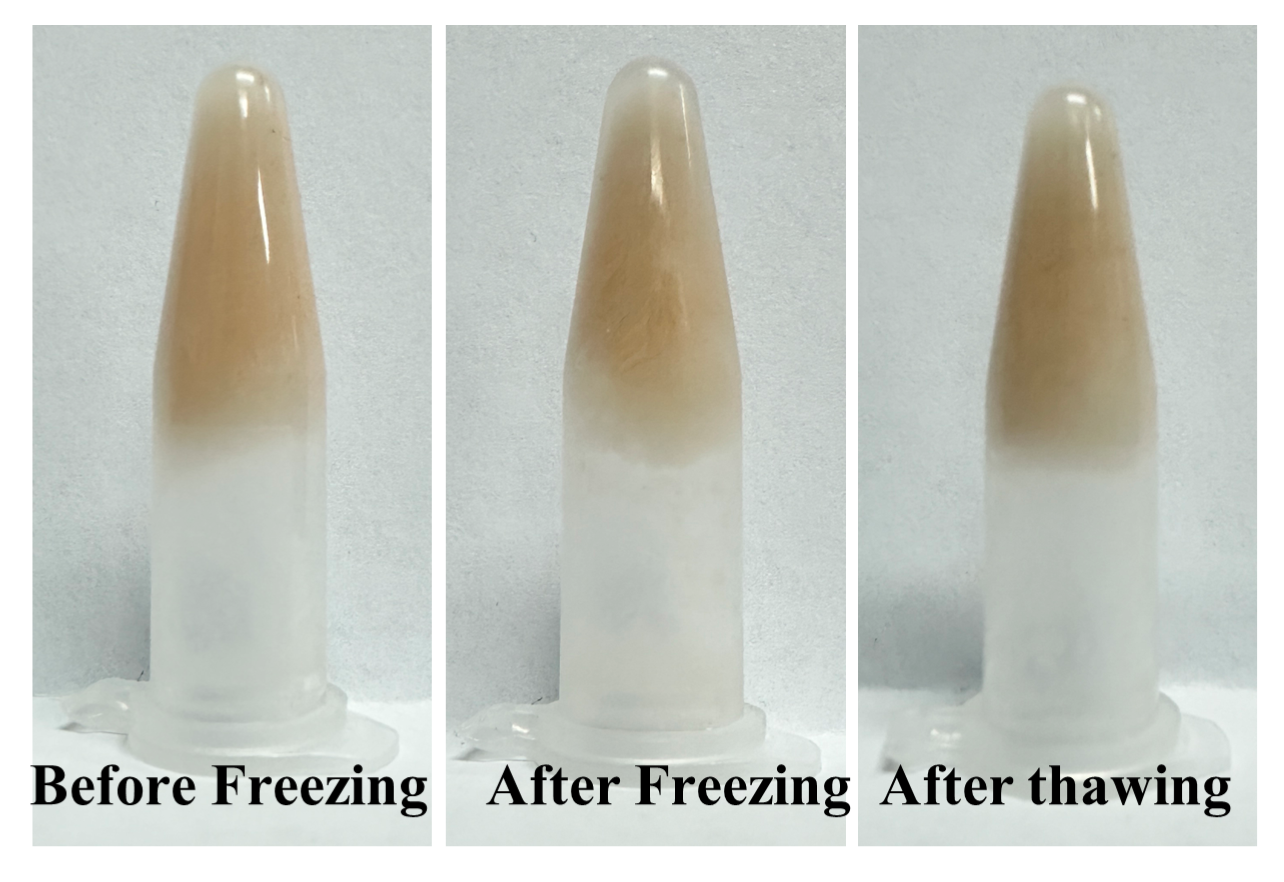


**Figure S4.** The state changes of BDN before and after freezing.


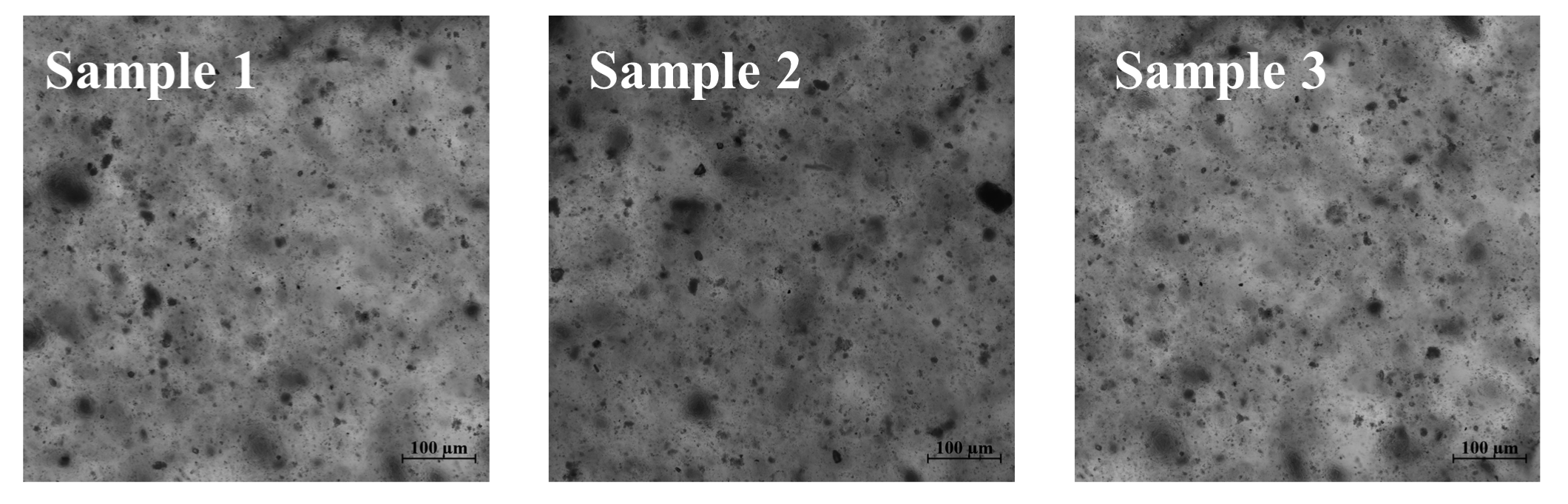


**Figure S5.** The confocal laser images of BDN smear.


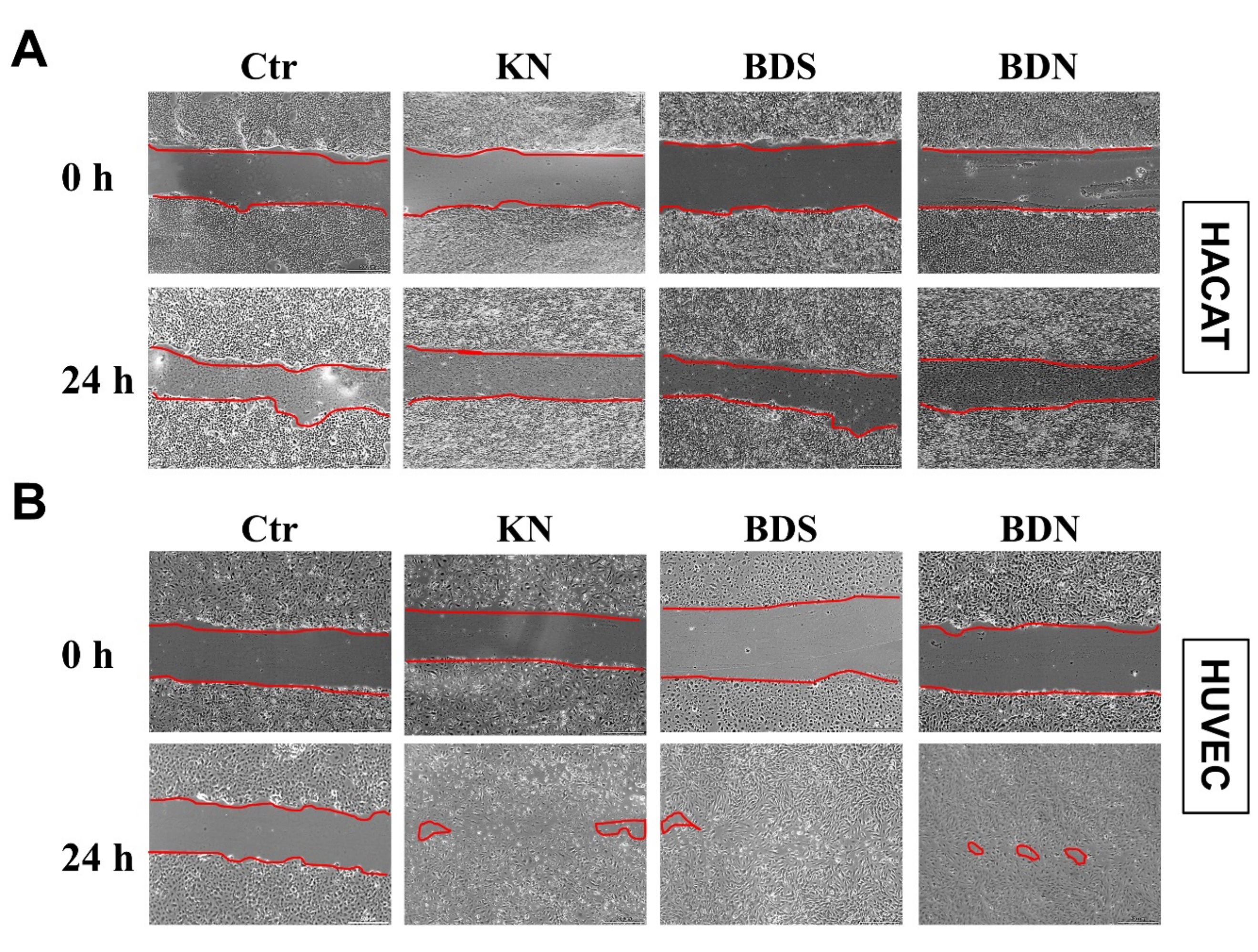


**Figure** **S6.** The influence of leachate from KN, BDS and BDN to the migration of (A) HACAT and (B) HUVEC cells with cell medium as a control.


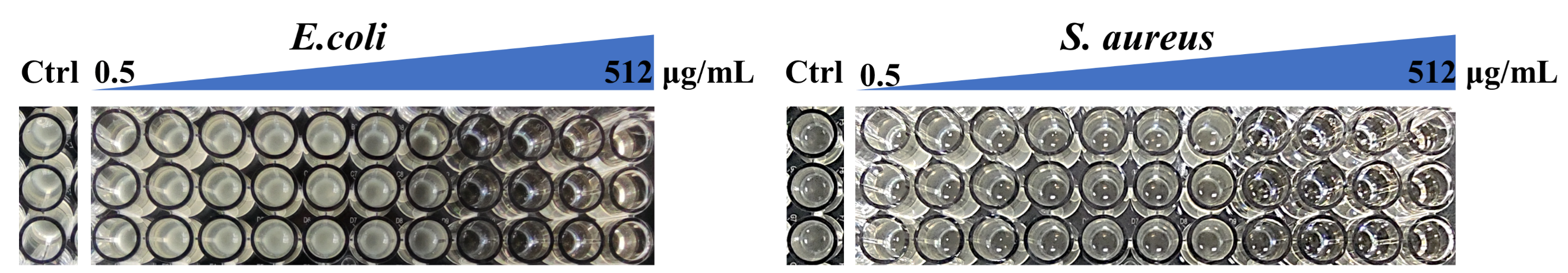


**Figure S7.** Photographs of medium cocultured bacteria with different concentrations of leachates from BDN.


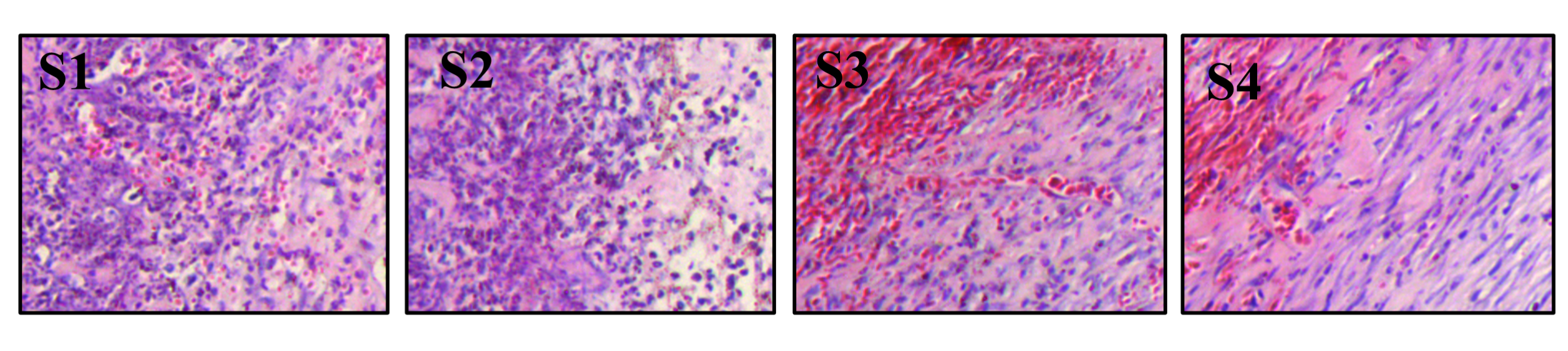


**Figure S8.** The magnified H&E staining images of various groups at day 14 in Figure 5.


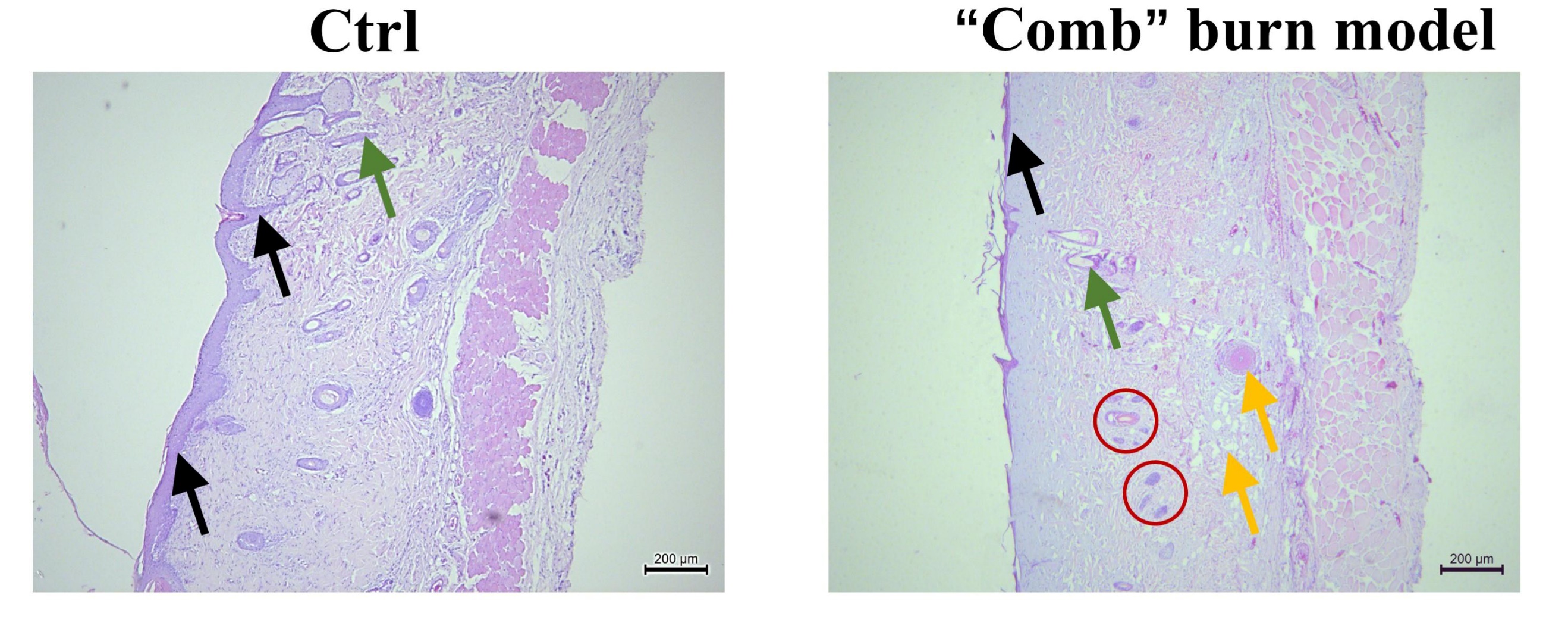


**Figure S9.** H&E staining of burn wounds of rats. The black arrow indicates epidermal histopathology, the green arrow indicates superficial dermis pathology, the yellow arrow indicates deep dermis pathology, and red circles indicate the residual skin appendages.

**
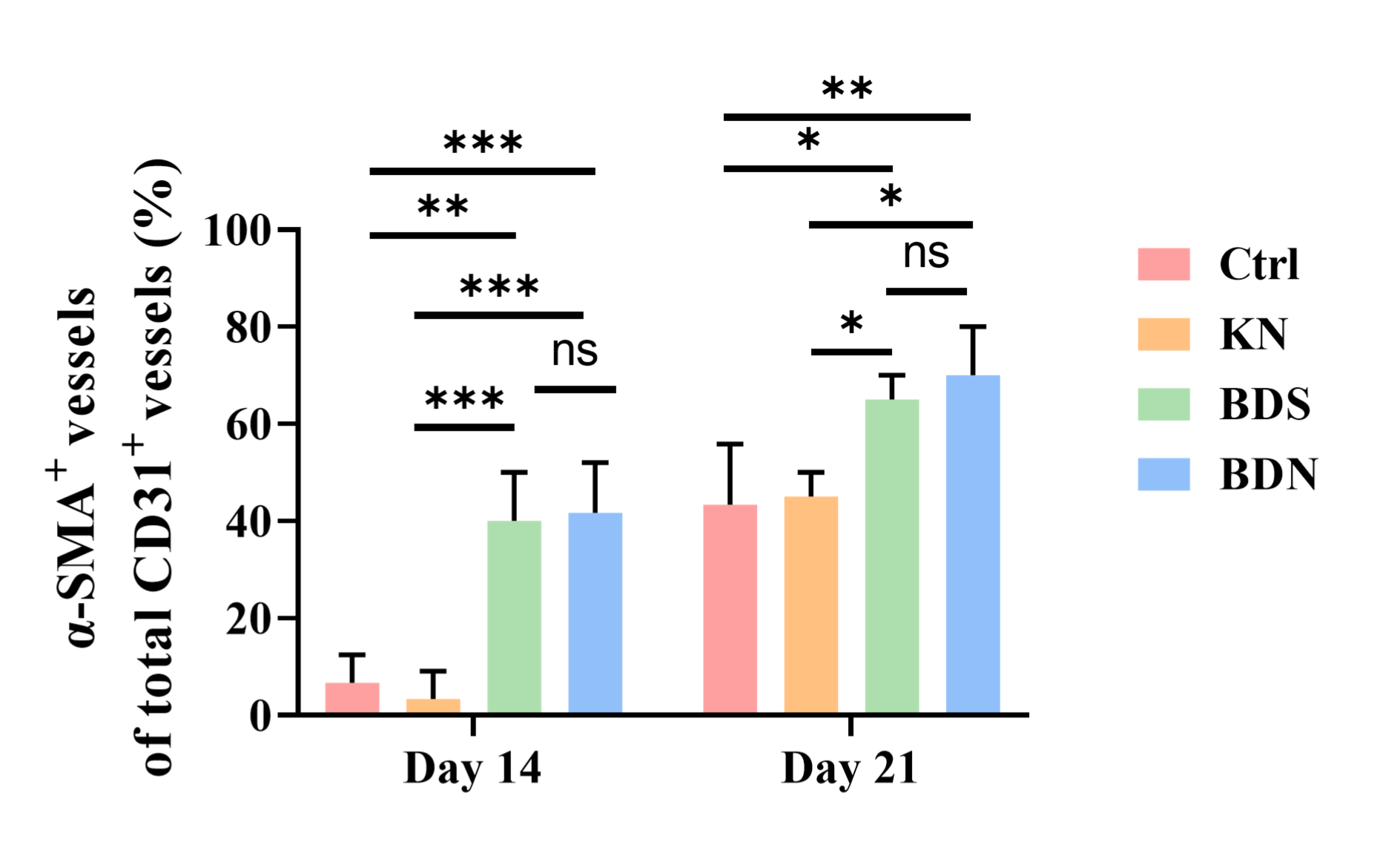
**

**Figure S10.** The statistical analysis data of *α*-SMA^+^ vessels of total CD31^+^ vessels after treated with various groups on a full-thickness wound healing of diabetic rat, n =3. All data are presented as mean ± SD. Data were analyzed using contrast analyses following one-way ANOVA. **p* < 0.05, ***p* < 0.01, ****p* < 0.001, *****p* < 0.0001. ns, No significant difference.


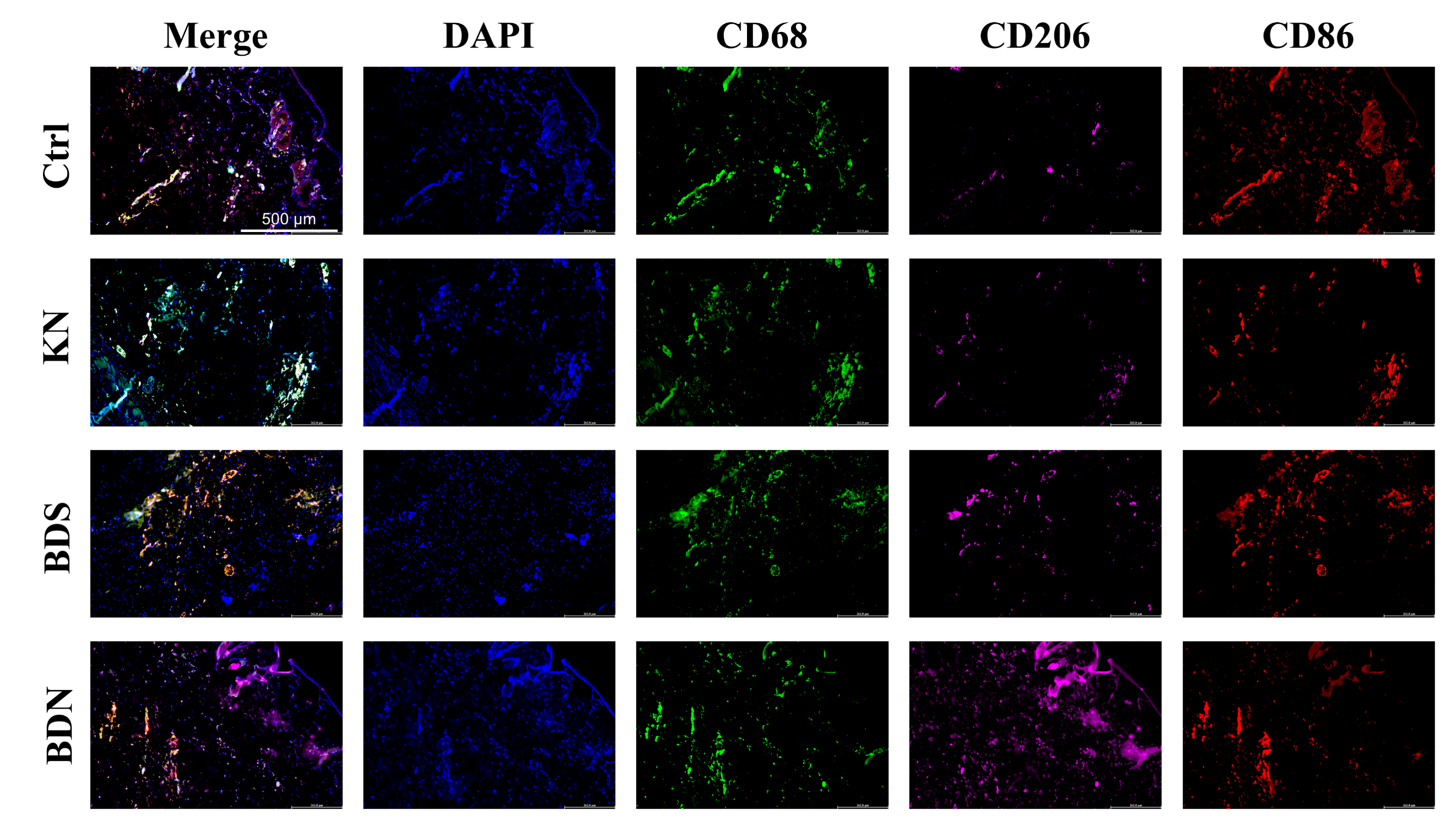


**Figure** **S11.** CD68, CD206 and CD86 immunofluorescence staining for each group at different time.


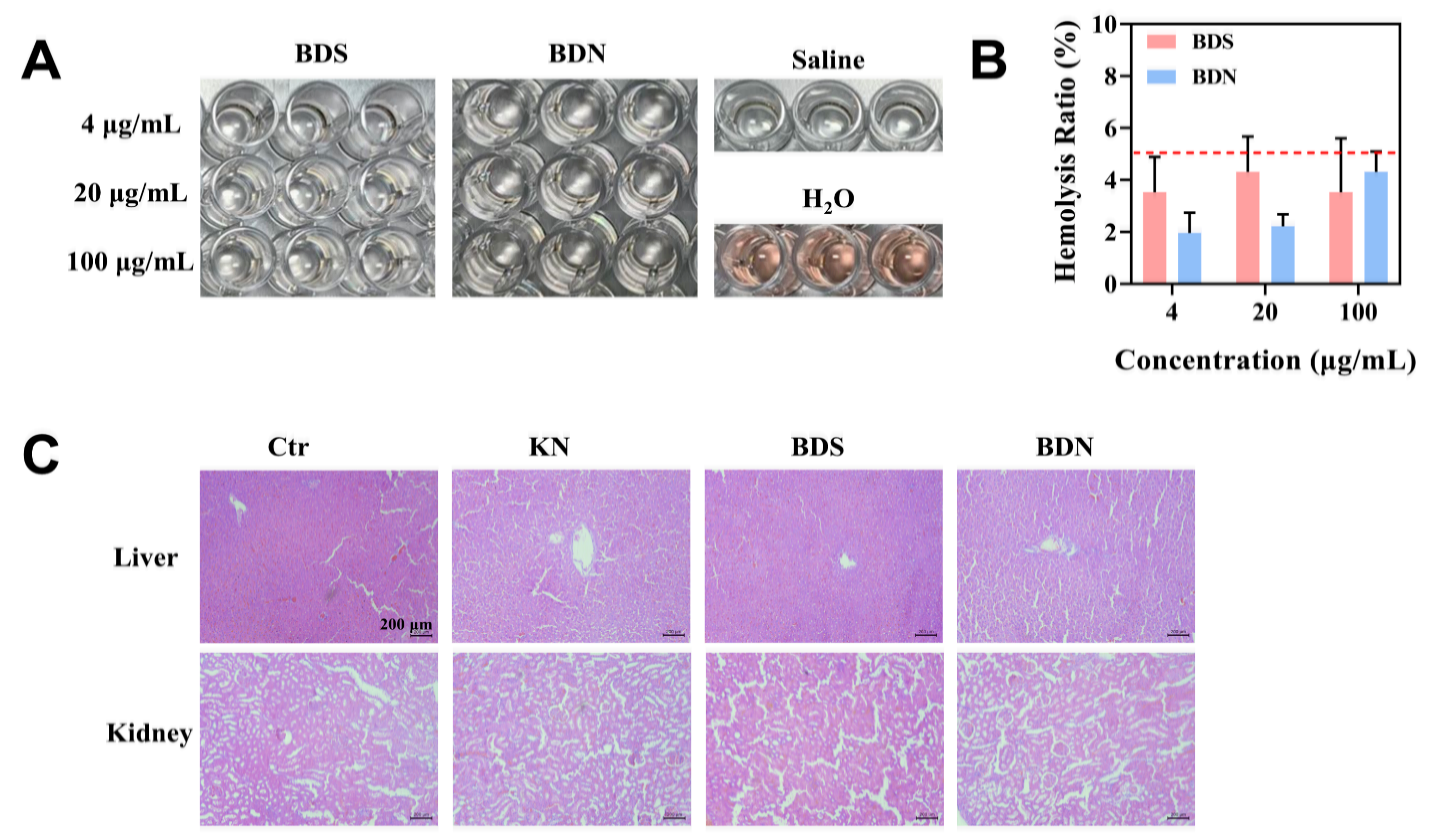


**Figure S12.** (A) Optical images of the hemolysis evaluation. Saline and pure water were used as the negative and positive controls, respectively. (B) Hemolysis ratio of different formations over various concentrations, n = 3. (C) H&E stained liver and kidney from the rat with different treatment.
